# GPathFinder: Identification of Ligand-Binding Pathways by a Multi-Objective Genetic Algorithm

**DOI:** 10.3390/ijms20133155

**Published:** 2019-06-28

**Authors:** José-Emilio Sánchez-Aparicio, Giuseppe Sciortino, Daniel Viladrich Herrmannsdoerfer, Pablo Orenes Chueca, Jaime Rodríguez-Guerra Pedregal, Jean-Didier Maréchal

**Affiliations:** Departament de Química, Universitat Autònoma de Barcelona, 08193 Cerdanyola del Vallès, Barcelona, Spain

**Keywords:** multi-objective genetic algorithm, molecular modeling, ligand diffusion, computational chemistry, molecular docking, drug design

## Abstract

Protein–ligand docking is a widely used method to generate solutions for the binding of a small molecule with its target in a short amount of time. However, these methods provide identification of physically sound protein–ligand complexes without a complete view of the binding process dynamics, which has been recognized to be a major discriminant in binding affinity and ligand selectivity. In this paper, a novel piece of open-source software to approach this problem is presented, called GPathFinder. It is built as an extension of the modular GaudiMM platform and is able to simulate ligand diffusion pathways at atomistic level. The method has been benchmarked on a set of 20 systems whose ligand-binding routes were studied by other computational tools or suggested from experimental “snapshots”. In all of this set, GPathFinder identifies those channels that were already reported in the literature. Interestingly, the low-energy pathways in some cases indicate novel possible binding routes. To show the usefulness of GPathFinder, the analysis of three case systems is reported. We believe that GPathFinder is a software solution with a good balance between accuracy and computational cost, and represents a step forward in extending protein–ligand docking capacities, with implications in several fields such as drug or enzyme design.

## 1. Introduction

The recognition mechanism between proteins and ligands is of crucial importance in many aspects of biological chemistry such as drug or enzyme design [1]. Computational approaches are key in these fields since they provide explicit molecular descriptions of the binding mechanism at the atomic scale. The modeling of protein–ligand interactions could be tackled from very different angles and a balance between the accuracy of the energetics and the size of the biochemical space to explore is fundamental to decide which method(s) to apply.

When looking for putative structures of stable protein–ligand complexes (e.g., identification of binding sites, virtual screening, etc.), the standard methodologies are based on protein–ligand dockings [2]. These methods use simplified force fields of the energy of interaction (scoring functions) [3] combined with explosive conformational explorative algorithms such as Genetic Algorithms (GAs) [4] or Monte Carlo [5]. When accurate binding energies are required (e.g., free energy of binding), methods based on accurate force field and extensive conformational sampling such as Molecular Dynamics (MD) are applied (i.e., thermodynamic integrations) [6,7]. In both cases, the geometric exploration of the protein–ligand interaction is mostly limited to the binding site of the ligand and a selective relaxation of the receptor.

The elucidation of structures of stable protein–ligand complexes does not necessarily accounts for the key determinants of protein–ligand interactions [8]. Indeed, the characterization of ligand pathways in proteins has been recognized to be a major discriminant in binding affinity and ligand selectivity [9]. These mechanisms are relatively slow in nature and their simulations by computational methods imply very large sampling. Elucidating ligand pathways could be fundamentally divided in three main families of techniques that can be sometimes combined together. The first one consists of looking for cavities that could connect the binding site to the bulk solvent without taking explicitly into account the ligand structure during the simulation process. These approaches could be applied to a standalone crystal structure, but accurate results are generally obtained from the analysis on MDs [10], some of them very large. Programs based on this approach are Caver, ChExVis, POCKET, VICE, CASTp, MOLE and MolAxis [10,11,12,13,14,15,16]. The second one consists of studying the ligand-protein interactions along a previously identified channel, simulating the diffusion of the ligand inside the cavity allowing more or less extensively the receptor to adapt. Metadynamics, steered MD, CaverDock and SLITHER are examples of this methodology [17,18,19,20]. The third consists of determining all possible channels taking the ligand explicitly into account as well as the adaption of the receptor. MoMALigPath, PELE, GRID-MD and ART-RRT are examples of software belonging to this family [21,22,23,24]. Whatever the approach, the accuracy of the energetic of the process is function of the quality of the sampling and the force field approximation used in the evaluation. In fact, in these approaches, the calculations of the molecular interactions could range from very simple geometric or energetic functions (i.e., clashes) to standard molecular mechanics force fields.

In this work, we present a novel software for the determination of ligand-binding paths in protein. The method combines full atom representation of the ligand and the protein with different levels of structural flexibility from small range (internal rotation, global rotation and translation of the ligand) to large range (rotameric exploration and normal mode analysis of the protein). It takes advantage of the multi-objective genetic algorithm engine of GaudiMM [25] to account for fast energetic evaluation that include a docking scoring function (i.e., Autodock Vina) and steric clashes. In the middle, between cavity search algorithm and extensive MD simulation, GPathFinder provides excellent results in a benchmark of 20 systems whose studies have been reported in the literature (representing the wider benchmark done so far on pathway determination software). Additionally, its usefulness is demonstrated on a detailed analysis on three systems: two well-characterized systems and one more where no mechanism of ligand binding has been proposed so far.

## 2. Results on Illustrative Cases and Discussion 

Aiming to show the capacities and accuracy of the results that can be obtained with GPathFinder, three cases have been chosen to study in detail. They have a variety of ligand (from 6 to 29 atoms) and protein (from 231 to 477 residues) sizes, and also different levels of previously known data to compare the results: from no knowledge about the concrete system (only about other members of the same family) to X-ray structures of different positions of the ligand along the binding process.

### 2.1. Transport of Glycerol Across Aquaporin

Aquaporins (AQPs) are a family of transmembrane tube-shaped proteins, allowing transportation of water and other small neutral molecules in and out of the cells. Present along all domains of life, they are of substantial biological importance in the metabolism of the organisms and are involved in several human diseases such as abnormalities of kidney function, loss of vision or onset of brain edema [26]. The first aquaporin was reported in 1993 by Agre and coworkers [27], and many others AQPs have been characterized since then.

Traditionally, AQPs have been classified into two main sub-families according to their function [28]: strict aquaporins, which only are selective to water, and aquaglyceroporins, which can transport water and other small molecules such as glycerol. A third class was added from the characterization of an archaeal aquaporin in 2003 [29], and a more complex classification has been recently proposed due to the latest incorporations to the AQPs family, with unexpected diversity [30].

**Figure 1:**
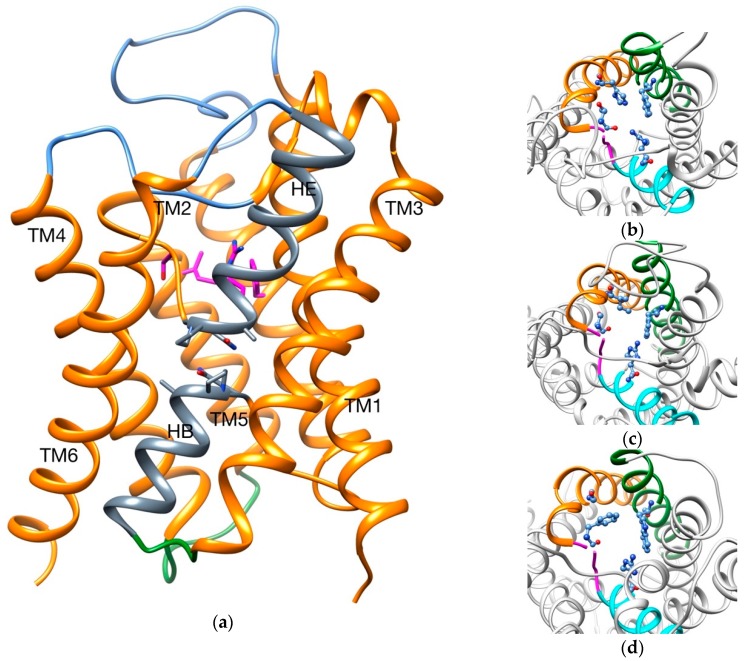
(**a**) Overall structure of an aquaporin. Extracellular loops are colored in blue, intracellular loops in green and the two hemihelices (HB and HE) in gray; SF is highlighted in magenta sticks and Asn-Pro-Ala motifs in gray sticks. In lateral panels, SF for different AQPs are shown: (**b**) AqpZ; (**c**) AqpM; (**d**) GlpF. TM2 is colored in green, TM5 in orange, HE in cyan and TM5-HE loop in magenta; SFs are highlighted in blue balls and sticks.

Despite this high diversity, the general tertiary and quaternary structures are retained throughout the family: six transmembrane domains (TMs), three extracellular and two intracellular loops, and two inverted hemihelices denominated HB and HE (Figure 1). 

How different AQPs can be selective to different permeants is believed to be a mix between size restriction of the channel and intermolecular interactions in determinate regions (two Asn-Pro-Ala motifs and the aromatic/arginine selectivity filter (SF)) [26,31,32,33]. For example, the narrowest point of the channel has a diameter of ~2 Å, ~2.5 Å and ~3.4 Å for AqpZ (aquaporin Z, strict aquaporin), AqpM (archaeal aquaporin) and GlpF (aquaglyceroporin), respectively [33,34,35]. Also, different residues compose the SF in each case (Table 1 and Figure 1b–d).

In this case study, the objective was to use GPathFinder to find out possible differences in terms of geometric constraints and binding energy profiles (with special interest in the SF region) when transporting glycerol across three AQPs known to be selective to different permeants (Table 1): AqpZs are only permeable to water, GlpFs are also permeable to glycerol (besides other small molecules) and AqpMs are in the mid-space, with a much lower permeability to glycerol than GlpFs—some authors [33] even inferred that glycerol could be ruled out from AqpM permeants.

A first set of calculations were run aiming to enlighten possible geometric constraints in the transport of glycerol. As initial and final points, the extracellular and intracellular regions were selected, respectively. Thus, the shape and length of the solutions proposed by GPathFinder are expected to be similar to each other. The optimization was made using only the geometric criterion trough average steric clashes evaluation. A second experiment was made centered on the SF vicinity: a glycerol was configured to cross the SF (a section of ~10 Å of the channel with the SF in the center was chosen). Besides the average steric clashes criterion, the binding energy (average Vina score of all frames) was taken into account in the pathway optimization. 

Ten runs per AQP were carried out with GPathFinder, and a pool of 100 pathways were obtained for each AQP in the first experiment. Two values have been analyzed (Table 2): the average volume overlap of all frames of the pathway and the volume overlap of that frame with highest value (meaning the bottleneck for glycerol along the trajectory). Average clashes along the trajectory are ~2.4 times higher for strict aquaporin than for aquaglyceroporin, leading to the conclusion that the overall geometric configuration of the channels could play a role in the glycerol permeability, what agrees with already reported data [26,32]. Archaeal aquaporin has an average value nearer to GlpF than to AqpZ, which is coherent with experimental data [33] that found glycerol molecules in all parts of the channel except on the SF region. Frames with highest clashes are always those with the ligand in the vicinity (distance <= 5 Å) of the SF residues, confirming the hypothesis that the geometric configuration of this zone takes an important paper on the permeability. Again, maximum values found for AqpM are nearer to GlpF than to AqpZ, in concordance with the hypothesis that the SF of AqpM could be permeable to glycerol if only geometric constraints are taken into account.

In the second experiment, two simultaneous objectives (clashes and Vina score) were minimized. As the resulting trajectories along the ten runs of calculation are quite similar in shape and length, a good method to analyze the results is to group all the solutions of each AQP study and extract their Pareto front. This selection leads to a total of 230 pathways (85 for AqpZ, 72 for AqpM and 73 for GlpF). 

**Figure 2:**
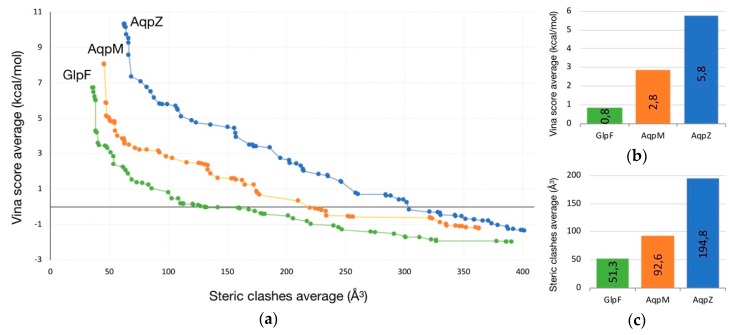
(**a**) Pareto front of solutions for different AQPs (GlpF in green, AqpM in orange, and AqpZ in blue). (**b**) Being fixed steric clashes to 100 Å^3^, Vina score for each AQP is represented. (**c**) Being fixed Vina score to 3 kcal/mol, steric clashes for each AQP is represented.

The analysis of the Pareto fronts plot (Figure 2) reveals a clear ranking in the pathway evaluation: those with best clashes-Vina relation are the GlpF solutions, followed by the ones for AqpM and being AqpZ the worst case. It leads to the following conclusions:

- Intermolecular interactions at the SF region (together with its geometric configuration) have a clear influence on the permeability/non-permeability to glycerol of GlpF/AqpZ.
- A significative difference is observed in the intermolecular interactions at the SF region of GlpF and AqpM, which can be relevant to explain their different glycerol diffusion rates.

### 2.2. Unbinding of a Suicide Inhibitor from hIDO1

Human indoleamine 2,3-dioxygenase 1 (hIDO1) is a heme-containing enzyme present in many tissues that catalyzes the dioxygenation of tryptophan. It is considered an important target for cancer immunotherapies, due to evidence that its inhibition could help to prevent the interference of hIDO1 with tumor cells function [38,39].

Between the inhibitors that have been developed for hIDO1 only one, BMS-986205 (BMS), acts as a suicide inhibitor. BMS inhibits the enzyme in a permanent manner by binding to the apo-form of hIDO1 at the heme location hence avoiding cofactor inclusion [40]. 

**Figure 3:**
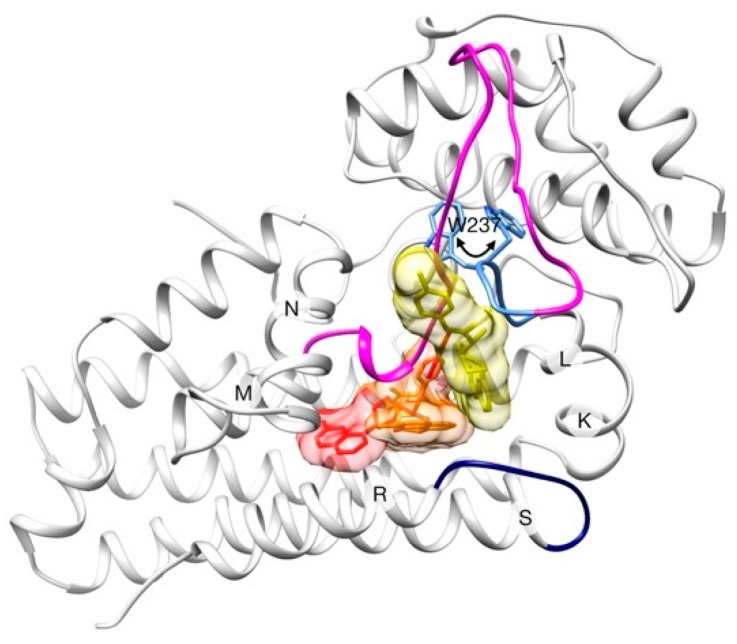
Schematic representation of the binding process of BMS to hIDO1. The three consecutive positions of the inhibitor are shown in yellow (step 1), orange (step 2) and red (step 3). Only one enzyme conformation (at step 3) is shown for clarity. LM-loop is colored in magenta (with its so-called “hairpin” in blue), and RS-loop is colored in dark blue. Rotation of W237 which produces the un/refolding of LM-loop hairpin is highlighted in sticks

A X-ray structure of hIDO1 was reported in 2005 [41], proposing the ligand/substrate entrance between helices K-L and N, near the RS-loop. A more recent study [42] characterized three X-ray structures that display different steps along the binding mechanism of the BMS inhibitor and thus proposes a clear binding pathway (Figure 3).

In this case study, we aim at finding binding pathways of the inhibitor and compare the results with the experimental interpretation. As seen before, BMS exhibits an unusual flexibility along the binding process, which together with the structural changes observed in the receptor, represent a significant challenge for ligand pathway predictors. A set of five runs were carried out starting the simulations with the inhibitor molecule at the heme binding site (PDB code 6mq6, chain B). No restrictions were imposed about the direction that the inhibitor has to follow to leave the enzyme. The optimization of the solutions was configured to take into account both steric clashes and binding energy.

**Figure 4:**
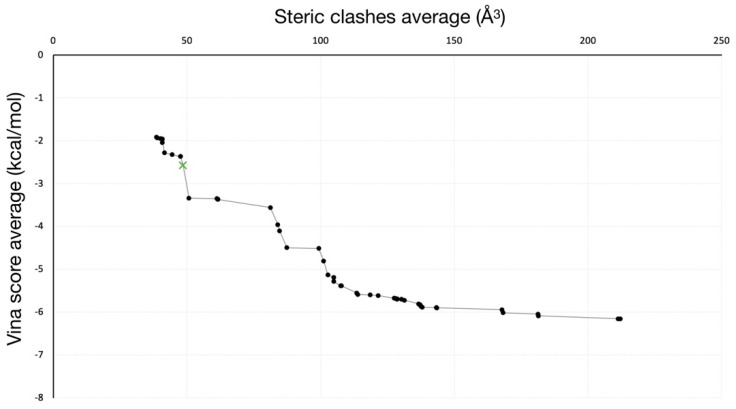
Pareto front of solutions obtained from GPathFinder. Solution selected for further analysis is colored in green with a “x” marker.

**Figure 5:**
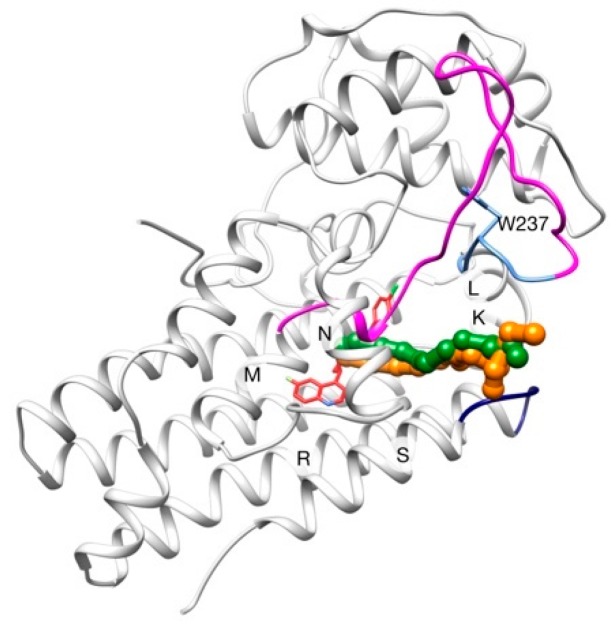
Pathways from GPathFinder calculations (colored in orange and green), where each point represents the BMS center of mass at this frame. LM-loop is colored in magenta, and its hairpin in blue (W237 in sticks). RS-loop is colored in dark blue and BMS in its binding pose is shown in red sticks.

A pool of 293 solutions was obtained from the calculations. Dominant solutions for both clashes and Vina scores were selected, leading to a total of 50 solutions that configure the Pareto front (Figure 4). Among them, two different morphologies (i.e., points that conform the trajectory) of pathways were identified (Figure 5), being the other 48 solutions with the same direction as the former but with differences in ligand or protein conformations in one/some of the frame/s.

In all these pathways, the entrance of the ligand is produced between helices K-L and N, making use of the hole near the RS-loop proposed in [41] rather than nearer to the LM-loop using the W237-switch mechanism. This preference could be explained because the incapacity of NMA to find a conformation with a displacement big enough in this concrete region (due to its global nature) or, even simpler, because all the solutions calculated with GPathFinder have a better score than those passing nearer W237, which were then discarded in the evolutive process of the algorithm. A further MD study could probably provide some insight on this issue, but that is out of the scope of the present work.

**Figure 6:**
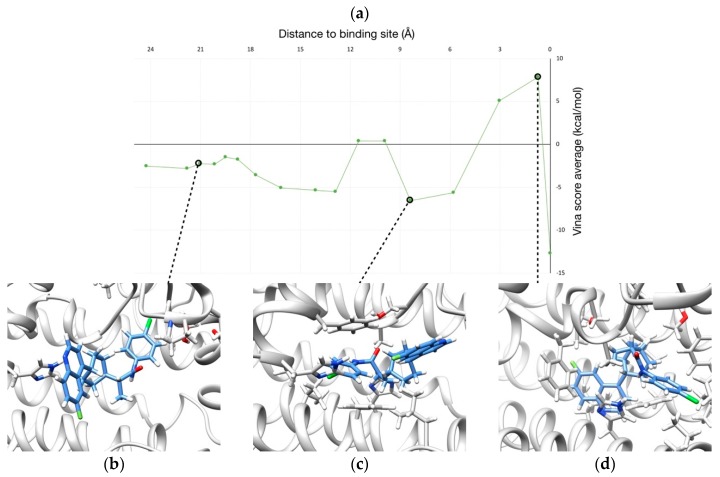
(**a**) Profile of binding energy (i.e., Vina score) along the binding pathway of BMS. (**b**–**d**) Three representative snapshots of the binding route are shown.

Solution with lower Vina score and average clashes under 50 Å^3^ was selected for further analysis (Figure 6a), revealing that BMS inhibitor adopts different conformations along its binding process (in a similar manner than “extended”, “kinked” and “bent” conformers found in [42]). It must be noted that hydrophobic interactions mainly represent the key recognition factor of the binding process in the analyzed pathway (Figure 6b–d).

### 2.3. Human Cytochrome P450 2C19

Cytochrome P450s (CYPs) are a large family of heme-containing enzymes with a wide variety of functions, including steroid, vitamin A and D, fatty acid and eicosanoid metabolism [43]. They are responsible for metabolizing a ~75% of the (small molecule) drugs available [44], being CYP2C19 one of the five with highest activity [45]. 

Although the overall secondary structure and folding are conserved along the family, there is significant structural variability in the active site and the access/egress routes [46]. For example, a comparison of the CYP2C19-0XV complex (PDB code 4gqs) and the CYP2C9-flurbiprofen complex (PDB code 1r9o) indicates deviations greater than 3.0 Å for equivalent Cα of the outer substrates of their binding cavities [47]. The 0XV refers to the compound (4-hydroxy-3,5-dimethylphenyl)(2-methyl-1-benzofuran-3-yl)methanone.

**Figure 7:**
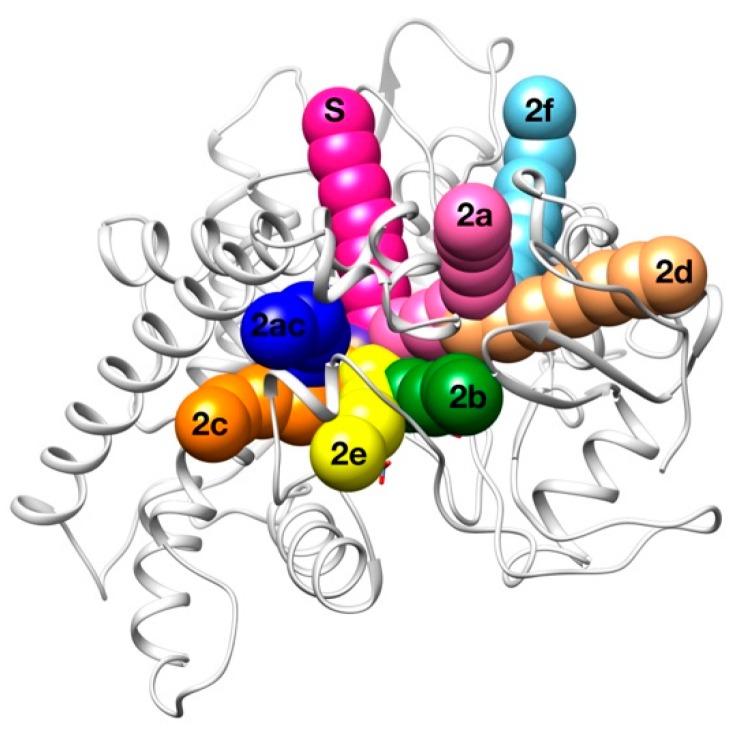
Schematic representation of the binding channels for P450.

Several (un)binding routes were identified in 2007 in a computational study using all the CYP structures available until then [48], defining also a nomenclature for the pathways which is used in this study (Figure 7). Depending on the concrete CYP and substrate combination (even for the same CYP and different substrates), it was found that the channel/s used differ; so it suggests that channels play an important role in substrate selectivity [46,49].

Here, we aimed to provide some insight on the channel/s used by 0XV to access the CYP2C19 active site, which, to the best of our knowledge, are still not characterized. 50 runs minimizing only steric clashes and 50 runs taking into account both geometric and binding energy criteria were carried out using the default parameters for the GPathFinder input file. The substrate molecule was placed in its binding position (PDB code 4gqs) and no restrictions about the direction to exit the protein were imposed.

In this case, results are similar in both experiments (Table 3), suggesting that the geometric constraints of the different routes are more important than intermolecular interactions in the selectivity of the 0XV by CYP2C19. The only exceptions are the appearance of the “2a” and “2b” routes (frequencies of 13.4% and 6.9%, respectively) when taking into account the Vina score in the evaluation. In the majority of the solutions, 0XV used the solvent channel to access the binding site, which is present in half of the P450s studied by Cojocaru et al. [48]. In second position, 0XV accesses trough channel 2c between the G and I helices and the B’ helix/BC-loop. Other routes observed with very minor frequency are the 2e and 2ac. The sum of these P450 well-characterized pathways represents more than the 95% of results in both studies, meaning that GPathFinder was able to find good results in a complex system using a standard input file.

## 3. Materials and Methods 

GPathFinder is designed as a novel extension of the molecular modeling platform GaudiMM, which is an implementation of the NSGA-II multi-objective genetic algorithm [50]. While the GA core is based on the Deap package [51], UCSF Chimera [52] provides the molecular framework upon which all the specific modules are built. This extension implements a set of new genes (named *path*, *path_torsion*, *path_normalmodes* and *path_rotamers*) and a new objective (i.e., *path_scoring*), among other minor improvements in the GA itself (actualization of the selection algorithm and performance). 

One of the most powerful features of GaudiMM is the clear separation between exploration (i.e., generating new solutions to the problem) and evaluation (i.e., selecting some of them according to appropriate evaluation functions). The iteration of this cycle (generate new solutions > evaluate these solutions > select the best ones) will eventually end with a pool of good-enough candidates to solve the proposed modeling problem.

**Figure 8:**
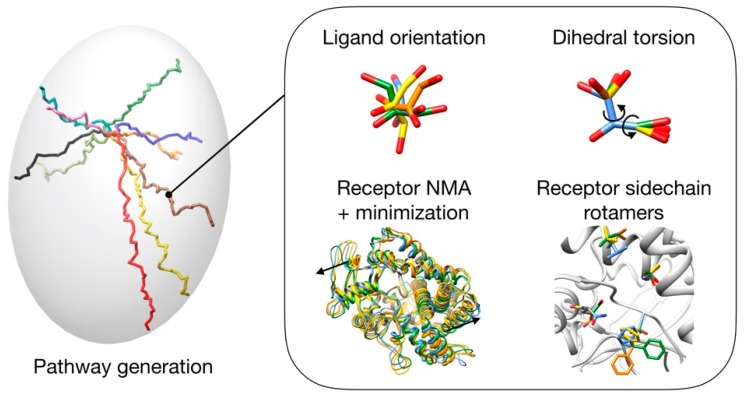
Scheme of different options (genes) that can be used to generate possible solutions.

GPathFinder takes advantage of this modular architecture and provides a new set of genes (in charge of the exploration, Figure 8) and objectives (controlling the evaluation) suitable to address the prediction of binding/unbinding trajectory of a ligand at atomistic level. The combination of several genes and objectives constitutes the so-called “recipe”, which takes the form of a .yaml input file [53].

### 3.1. Pathway Generation

A pathway, by definition, is a set of points between a start and an end that draws a trajectory. We call frame the concrete conformation and relative position of both the ligand and the receptor molecules at one point of the pathway. 

How and where the ligand is placed along the pathway is the main function of the *path* gene. There are three options that the user can configure (Table 4), which allow the following calculations:

1. Unbinding trajectories knowing the initial point (i.e., binding pose).
2. Binding trajectories starting from the six ends of the inertia axes of the protein and finishing in a known active site.
3. Possible pathways between previously stablished initial and final points.

**Figure 9:**
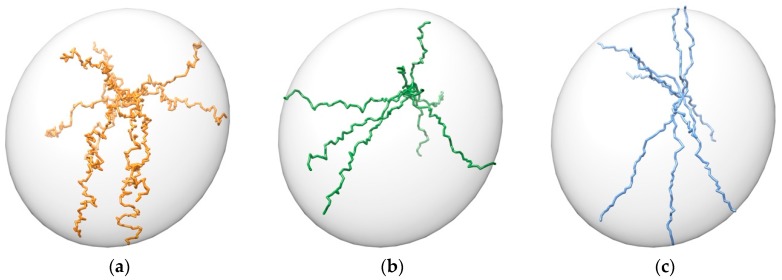
Different shapes of pathways. Maximum distance between points is set to 1 Å in all cases. Minimum increment is set to: (**a**) −0.4 Å; (**b**) 0.0 Å; (**c**) 0.8 Å. 10 random trajectories from a point inside the ellipsoid towards its exterior are shown for each case.

The binding trajectory of a ligand can take a wide variety of shapes: from an almost straight line to a very curved path “U”, “L” or “S” shaped. To ensure that GPathFinder can address this diversity, the minimum distance increment from the origin point of each frame can be configured and adapted to the concrete system—by default is 0.8 Å, considering it is a good value for a general case—(see GA parameter set up). The lower the value, the more curved can be the solutions (Figure 9), but also a higher number of frames will be generated, with an associated increment of computational cost.

To avoid unfairly crossing barriers such as helices or beta strands, the maximum distance between the ligand center of mass in two consecutive frames is automatically set by the program in function of the ligand size (although it can also be changed by the user).

Free relative orientation of the ligand is allowed at each point of the pathway, and its flexibility is considered by free rotation of dihedral bonds (i.e., *path_torsion* gene). Types or names of the atoms which bonds are allowed to rotate can be configured by the user (by default, all bonds containing non-terminal atoms and at least one atom of Chimera IDATM Type [54] C3, N3, C2, N2, or P are considered to be rotatable).

Considering the receptor, two levels of flexibility, global and local, are taken into account. ProDy implementation [55] of Normal Modes Analysis (NMA), a widely used method to model protein deformations induced by ligand binding [56,57,58,59,60], is in charge of generating the global protein conformations (i.e., *path_normalmodes* gene), which can be object of a further minimization by the OpenMM [61] engine (more details are provided in Supplementary Materials [62,63,64,65]). On the other hand, local exploration of the side chains flexibility on the vicinity of the ligand position at each frame is carried out by the *path_rotamers* gene, based on Dunbrack or Dynameomics rotamer libraries [66,67].

### 3.2. Pathway Evaluation

**Figure 10:**
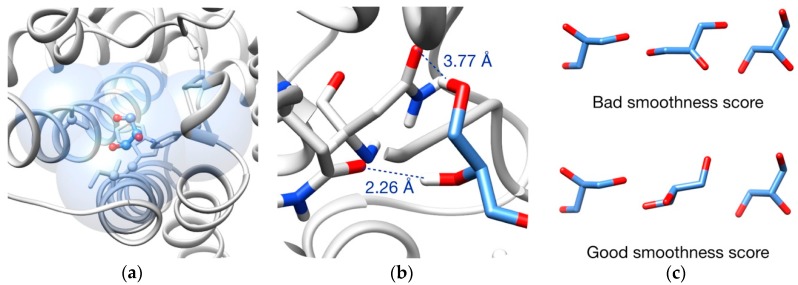
Pathway evaluation: (**a**) Example of clashes evaluation. Blue volume indicates the evaluation zone, which is formed by all atoms in a radius of 5 Å from every ligand atoms and carbons beta of the rotamers (highlighted in ball and sticks); (**b**) Example of Vina scoring. Only intermolecular interactions are taken into account. In dash lines, two interactions between ligand (blue sticks) and surrounding residues (gray sticks); (**c**) Example of smoothness evaluation. A bad scoring (ligand is flipped in the second frame) is shown at the top and a good scoring is shown at the bottom.

All the evaluation is implemented in the new *path_scoring* objective, and two basic metrics can be employed to determine the quality of a frame depending on the nature of the experiment. On the one hand, steric intra- and intermolecular clashes minimization can be used to obtain geometrically feasible pathways. Taking as reference the ligand atoms and beta carbons of the surrounding rotamers, all the atoms in a radius of 5 Å from these reference atoms (Figure 10a) are evaluated in terms of volumetric overlap (a complete definition is provided in Supplementary Materials) [68].

On the other hand, the binding energy can be optimized by minimizing its Vina score [69]. This allows the obtaining of an energetic profile of the pathways proposed by the program. It has to be mentioned that this second approach only considers intermolecular interactions (Figure 10b), so it has to be combined with a clashes evaluation to avoid unrealistic conformations of the ligand and overlapping between rotamers.

As a complement, a “smoothness” scoring objective can be added if the user is interested in obtaining trajectories where the ligand movements are smooth between two consecutive frames (Figure 10c), meaning to avoid flipping or big conformational changes. This is achieved by minimizing the RMSD (Root-Mean-Square Deviation) of two consecutive ligand conformations (a cutoff RMSD can be set to let some degree of permissiveness).

Once the frame evaluations are obtained, the final score of the pathway will be the average or maximum of them (a parameter set by the user controls which method is used). For example, if it is previously known that the algorithm is dealing with similar-shaped pathways (e.g., illustrative case of aquaporins), the best option would be to optimize the average score. Instead, if the shape or length of the trajectories are previously unknown or expected to have a high diversity, the best option would be to reduce it at the maximum to avoid distortions due to the average calculation (e.g., a pathway that crosses a beta-strand in a frame but where the rest is low-scored would be considered better on average than others that always transit by fair but assumable scored frames).

### 3.3. Pathway Refinement

Although GPathFinder generates pathways where the ligand positions are close enough to avoid unfair movements, bottlenecks may still be possible between two consecutive frames. For cases where a real continuum trajectory may be necessary, a so-called “pathway refinement” script is provided. An improvement of the Rapidly exploring Random Tree (RRT) [70,71] called RRT-Connect [72], which has proven particularly effective at finding a path between a fixed start and end configuration, is the basis for this implementation. Further details are provided in Supplementary Materials, including the algorithm specifications in Table S1.

### 3.4. Set up of the GA Parameters

One of the difficulties when working with GAs is the correct set up of its different parameters. In the case of NSGA-II, the number of generations and the population size have to be provided in advance. Based on the experience, a population of 12 individuals and several generations between 500 and 1000 are good values to obtain a good balance between the quality of the results and the computation time.

In addition, the ratio of the two operators for generating the solutions (i.e., crossover and mutation) has to be proportioned by the user. Finally, in the case of GPathFinder, the parameter “minimum distance increment from the origin” of the path gene has an important role in the shape of the obtained solutions.

To set up these two latter parameters, a set of 40 ligand-receptor systems has been used as benchmark (Table S2). The selection was made aiming to preserve a wide diversity in terms of ligand and receptor sizes, function of the system, and apparent difficulty of the channels. Five values from 0.8 Å to -0.4 Å for “minimum distance increment from the origin” were tested (ten runs per system and value were launched optimizing only steric clashes). Three additional cycles of calculations with different ratios of the genetic operators were carried out aiming to achieve a consensus value for this parameter. After analyzing the results (Table S3), the better parameters were fixed as default (Table 5). 

### 3.5. Benchmark

A subset of 20 systems (Table S4) was selected for benchmark the quality of the solutions provided by GPathFinder. While maintaining the diversity, all of them have data available about the ligand diffusion channel/s, obtained either by computational [10,21,22,23,24,73,74,75,76,77,78,79,80] or experimental [41,42,75,81,82,83,84] methods. Twenty runs per system were carried out, optimizing steric clashes and Vina score simultaneously.

The receptor and ligand .mol2 files were prepared by the following operations in the PDB (Protein Data Bank) structure:

1. If necessary, select one of the chains.
2. Remove waters and other non-proteic molecules.
3. Remove alternative for side chains rotamers.
4. Add hydrogen atoms with UCSF Chimera “addh” command (necessary for Vina scoring).
5. Separate into two .mol2 files the ligand and the receptor molecules.

When non-standard residues are present (e.g., heme groups), they were properly parametrized [85]. Details about the parametrization and .yaml files for all the experiments (GA set up, benchmark and illustrative cases) are provided in Supplementary Materials. Home-made scripts, provided together with the source code of the program, were used to analyze the results.

**Figure 11:**
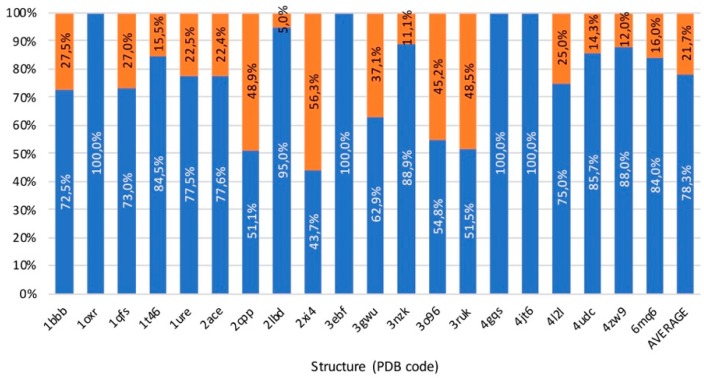
Summary of benchmark results. In blue, percentage of solutions that belong to already referenced pathways. In orange, percentage of solutions that belong to other pathways.

Results obtained from the benchmark, summarized in Figure 11 and detailed in Table S4, confirm the excellent capacity of GPathFinder to find solutions according to the existing literature in all the 20 systems studied. To make a correct reading of the results, it is fundamental to understand that GPathFinder find in the low-energy solutions all the pathways determined by previous studies of the systems of the benchmark set. The percentage of pathways belonging to already known routes is 78.3% on average, meaning that GPathFinder also finds novel pathways. It is important to notice that solutions included in ca. 22 % of novel solutions are not necessarily wrong neither meaningless since most appear extremely reasonable. However, for the sake of the benchmarking exercise we did not analyze all these systems and re-interpret previous studies one by one but decided to focus only on two systems of the set and apply our algorithm to a new one.

### 3.6. Usability, Availability, and Computational Cost

GPathFinder, as its root GaudiMM, is an open-source program licensed under Apache License v.2.0, meaning that the user has the freedom to use the software for any purpose, to distribute it, to modify it, and to distribute modified versions of the software, under the terms of the license. The source code and its documentation, example input files, refinement script and home-made scripts used in this article to analyze the results will be available free of charge from the date of publication at https://github.com/insilichem/gpathfinder.

The software is compatible with Linux and MacOS, and its main dependencies are: Python, UCSF Chimera, YAML, DEAP, ProDy, and AutoDock Vina. All dependencies (a complete list is provided in Supplementary Materials) are installed automatically, with the exception of UCSF Chimera, which has to be installed beforehand by the user. 

In all GA-based programs, the parametrization of the algorithm is one of the most cumbersome aspects. GPathFinder only requires a plain text .yaml file, which allows configuration of all these parameters besides the description of the system. Some example input files with a default parametrization are provided to facilitate this task.

Regarding computational cost, all GPathFinder calculations of this article were performed on a standard personal computer, with an intel core i7 processor and 8 GB of memory. Time of computation ranges from 30 min to 6 h depending on the number of objectives selected, the parametrization of the GA and the size of the system. As GaudiMM calculations are run on a single core, several runs can be carried out at the same time depending of the number of cores of the computer (in our case eight cores). 

## 4. Conclusions

When we set up GaudiMM, our objective was to provide a genetic algorithm platform with a high adaptability and allow application to very different subjects. Its strength is the possibility for the user to set up his/her recipe for the system under study, defining which degree of conformational exploration is necessary and which combination of structural and energetic descriptors is relevant. GPathFinder is the second implementation that comes out of it. In the middle, between protein–ligand dockings and large-scale sampling methodologies, we believe the algorithm provides an interesting platform for the entire community dedicated to protein–ligand interactions. In fact, in a recent published work, an earlier version of the code presented here was applied to the discovery of an exo-hydrolase catalytic mechanism [86]. Further works along this line include providing semi-local structural rearrangement (i.e., small loop folding and unfolding upon binding), simulation of metalloligand binding path to biological hosts (i.e., metallic cofactor) [87], and the development of graphical interfaces to set up calculations. 

## Figures and Tables

**Table 1:** Summary of aquaporin structures used for the calculations.

PDB Code	AQP Subfamily	Permeant/s	Residues of the SF	Reference/s
1rc2 (a)	Strict aquaporin (AqpZ)	Water	F43, H174, T183, R189	[34]
2f2b (a)	Archaeal aquaporin (AqpM)	Water, glycerol ^1^	F62, I187, S196, R202	[29,33]
1ldi (a)	Aquaglyceroporin (GlpF)	Water, glycerol, urea, antimonite, arsenite, polyols, lactate	W48, G191, F200, R206	[36,37]

^1^ Much lower glycerol permeability than GlpF [29].

**Table 2:** Steric clashes for the three types of aquaporins (100 solutions were obtained for each one). Mean values for these 100 solutions and standard deviations are reported.

AQP	Considering the Frame with Highest Steric Clashes	Considering the Average Steric Clashes of All Frames
AqpZ	130.70 ± 27.92 Å^3^	23.95 ± 3.31 Å^3^
AqpM	95.63 ± 23.99 Å^3^	14.62 ± 2.45 Å^3^
GlpF	83.03 ± 18.85 Å^3^	10.19 ± 2.59 Å^3^

**Table 3:** Frequency of the different channels used by 0XV to access the binding site in CYP2C19. Percentage of solutions for each route with respect to the total of 50 runs is reported.

Access Channel	Frequency for Clashes Evaluation (%)	Frequency for Clashes + Vina Evaluation (%)
Solvent	62.0 %	49.2 %
2a	0.0 %	13.4 %
2b	0.0 %	6.9 %
2c	28.0 %	19.4 %
2e	6.0 %	2.0 %
2ac	2.0 %	4.5 %
Others	2.0 %	4.6 %

**Table 4:** Summary of available options to configure direction of the pathways in GPathFinder.

Option	Initial Point	Final Point	Function	Parameters of Path Gene
1	Known	Unknown	Unbinding trajectories	No necessity to configure if the ligand is positioned at the binding site
2	Unknow	Known	Binding trajectories	Destination (binding site coordinates)
3	Known	Known	Trajectories between two points	Origin (starting point) Destination (ending point)

**Table 5:** Default values for the genetic algorithm parameters.

Parameter	Default Value
Number of generations	500 (one objective) 750 (two objectives) 1000 (three objectives)
Population size	12 individuals
Minimum increment distance from the origin	0.8 Å
Proportion of crossover	0.2
Proportion of mutation	0.8

## Supplementary Materials

Supplementary materials can be found at https://www.mdpi.com/1422-0067/20/13/3155/s1.

## Abbreviations

0XV	(4-hydroxy-3,5-dimethylphenyl)(2-methyl-1-benzofuran-3-yl)methanone
AQP	Aquaporin
AqpM	Archaeal Aquaporin
AqpZ	Aquaporin Z
BMS	BMS-986205 inhibitor
CO	Carbon Monoxide
COHb	Carboxyhemoglobin
CYP	Cytochrome P450
GA	Genetic Algorithm
GlpF	Aquaglyceroporin
Hb	Hemoglobin
hIDO1	Human indoleamine 2,3-dioxygenase 1
MD	Molecular Dynamics
NMA	Normal Mode Analysis
PDB	Protein Data Bank
RMSD	Root-Mean-Square Deviation
RRT	Rapidly exploring Random Tree
SF	Selective Filter
TM	Transmembrane Domain
