# Supplementary material for: GPathFinder: Identification of Ligand-Binding Pathways by a Multi-Objective Genetic Algorithm
Source: Int J Mol Sci. 2019 Jun 28;20(13):3155. doi: 10.3390/ijms20133155 (PMC6651367; doi:10.3390/ijms20133155)
Supplement: Supplementary file 1 [file ijms-20-03155-s001.pdf]

International Journal of *Molecular Sciences*

Supplementary Materials

GPathFinder: identification of ligand binding pathways by a multi-objective genetic algorithm

José-Emilio Sánchez-Aparicio [1], Giuseppe Sciortino [1], Daniel Viladrich Herrmannsdoerfer [1], Pablo Orenes Chueca [1], Jaime Rodríguez-Guerra Pedregal [1] and Jean-Didier Maréchal [1,*]

[1] Departament de Química, Universitat Autònoma de Barcelona, 08193 Cerdanyola del Vallès, Barcelona, Spain; joseemilio.sanchez@uab.cat (J.-E.S.-A.); daniel.viladrich@e-campus.uab.cat (D.V.H.); pablo.orenes7@gmail.com (P.O.C), jaime.rogue@gmail.com (J.R.-G.P.), jeandidier.marechal@uab.cat (J.-D.M.), giuseppe.sciortino@uab.cat (G.S.)

* Correspondence: jeandidier.marechal@uab.cat

1. Computational details

1.1. Normal Modes Analysis (NMA) and energy minimization

ProDy [1] Rotation Translation Blocks (RTB) or Anisotropic Network Model (ANM) classes are used to generate global perturbations in the protein structure. The following parameters can be modified by the user to customize and adapt the resulting samples:

- "modes": number of non-zero eigenvalues/vectors to calculate (by default, the 20 first modes are calculated).
- "rmsd": average RMSD that the conformations will have with respect to the initial conformation (default is 2.0 Å).
- "group_by": calculate modes grouping atoms by residues, mass or use an all-atom model (either using alpha-carbons or all atoms). In the two first cases RTB ProDy class is used, while ANM is used in the latter (default is group by residues).

With this configuration, a pool of "n_samples" (default to 100) protein structures is generated by the ProDy engine at the beginning of the GPathFinder calculation. These structures can be submitted (if the user sets the parameter "minimize" to True) to a cycle of minimization iterations by the OpenMM engine [2]. In this case, the AMBER99SB force field [3] is used for standard residues (the user can provide its own parametrization if needed), and the minimization_tolerance and minimization_iterations parameters are set by default to 10 and 1000 respectively. The pool of protein conformations obtained after this NMA + (optional) minimization is used in the pathway generation as possible structures of the receptor.

1.2. Steric clashes evaluation

The process to evaluate the clashes score of a frame is:

1. Take all the ligand atoms and the beta carbons of all the surrounding residues.
2. Define a sphere with a radius of 5 Å from each one of these atoms. The conjunction of these spheres will constitute the evaluation zone.
3. All the atoms present in the evaluation zone are selected.
4. Detect clashes among all the atom pairs.
5. Evaluate the volumetric overlap in those atom pairs that have been clash-detected.
6. Sum all the volumetric overlaps of the search zone, which will be the clash score of the frame.

Steric clashes detection is made using the UCSF Chimera [4] interatomic clashes finder, which is based on Van der Waals radii (r_{VDW}) and user-specified criteria. In this context, a clash is defined as unfavorable interactions when atoms are too close together, considering that there is a clash between two atoms *i* and *j* when their *overlap* is >= *cutoff*:

$$overlap_{ij} = r_{VDWi} + r_{VDWj} - d_{ij} - allowance_{ij}$$

where allowance is a parameter to consider potential hydrogen bonds, being only applied in donor-acceptor pairs. A value of 0.2 Å is set for this parameter in GPathFinder, following the recommendations of UCSF Chimera documentation. The cutoff value can be parametrized by the user, but it is set by default to 0.6 Å, also following the UCSF Chimera recommendations.

The volumetric overlap of the atom pair [5], when a clash is detected by the previous defined method, constitutes the contribution of this pair to the final evaluation of the frame.

1.3. Parametrization in cases involving non-standard residues

The AMBER14SB force field [3] was used for standard residues, while the GAFF force field was adopted for the remaining atoms. Metal-bonding force constants and equilibrium parameters were obtained through the Seminario method, using Gaussian09 [6] to compute the geometry and harmonic frequencies at DFT level with the B3LYP3 functional combined with scalar-relativistic Stuttgart–Dresden SDD pseudopotential and its associated double-ζ basis plus a set of *f* polarization functions [7] for the metal. The 6-31G(d,p) basis set was used for H, C, O, N and S. Point charges were derived using the RESP [8] (Restrained ElectroStatic Potential) model. The force field building operations were carried out using the MCPB.py [9].

1.4. Pathway refinement

The findings of path planning algorithms used in robotics research can be applied to unbinding problems in biophysics and, in particular, Rapidly-exploring Random Trees [10] have proven to be well suited in crowded environments and efficient exploration of high dimensional configuration spaces given the number of torsional degrees of freedom of biomolecules [11,12].

A path tree is made up of nodes that contain the coordinate vector in configuration space and a reference to its parent node. This allows to construct a path from any node of the tree to the initial/final configuration by repeatedly looking up its parent node.

The RRT-Connect algorithm is given in Listing 1 and can be summarized in the following way: one of the two trees is expanded towards a randomly sampled node q_{rand} by finding the tree's nearest point q_{near} to it and taking a step ε from q_{near} in the direction of q_{rand}. If the new node q_{new} passes the COLLISION test, it is added to the tree, such that q_{near} becomes the parent node of q_{new}. Now the tree will expand further from q_{new}, but in the direction of the nearest node of the opposite tree, until it either collides or reaches it. In the first case, after a collision we swap the tree's roles and repeat the process, but if the trees connect, the unique path is constructed by successively looking up the parent nodes of both connection nodes.

Listing 1. RRT-Connect algorithm.

```
input: frame input nodes q_i, q_f
output: path

tree_i=[q_i]
tree_f=[q_f]
for i in 1..max_iterations
   q_random.x=RANDOM()
   q_near.x=NEAREST(q_random.x,tree_i)
   q_new.x=EXTEND(q_near.x,q_random.x)
   q_guide=NEAREST(q_new.x,tree_f)
   while COLLISION(q_new.x)=False:
      q_new.parent=q_near
      tree_i.append(q_new)
      q_near=q_new
      q_new.x=EXTEND(q_near.x,q_guide.x)
      if DIST(q_new.x,q_guide.x)<expand_dist:
         Return tree_i , tree_f
   SWAP(tree_i,tree_f)
path=MAKEPATH(tree_i,tree_f)
```

For our purposes the RRT-connect algorithm has been modified in the following ways, in accordance with improvements of the RRT algorithm applied to ligand unbinding [11]. These are summarized in Table S1.

Table S1. Algorithm specifications.

Type	RRT-connect
Metric	Weighted euclidean
Greed	10%
Expansion step	1/30th of the frame distance
Nearest node	Random pick out of the nearest 1%

The expansion step ε is set to 1/30th of the distance of the two consecutive frames to be refined. The NEAREST function chooses a random node within the nearest 1%. This has been shown to notably reduce the number of iterations needed because it avoids expanding towards nearer but bottlenecked nodes repeatedly. RRT algorithms are known to be sensitive to the chosen distance metric. In our case we have used a weighted euclidean metric over the configuration vector in order to give equal value to every component in the configuration space independently of its units. The greed was set to 10%, since the CONNECT function is very greedy in and of itself.

1.5. Software dependencies

GPathFinder relies on several dependencies, which are installed together with the software. The only exception is UCSF Chimera, that should be installed previously by the user. The complete list of dependencies is:

```
# Base dependencies
- python 2.7.*
- nomkl
- munch 2.2.*
- voluptuous 0.10.*
- click 6.7.*
- boltons 17.2.*
- deap 1.2.*
- pyyaml
- pychimera >=0.2.6
# Objectives and genes
- openmm
- openmoltools
- pdbfixer
- cclib
- autodock-vina
- autodocktools-prepare
- prody 1.8.*
```

2. Set up of GA parameters and benchmark

Table S2. Set of 40 ligand-receptor structures selected for calibration of the genetic algorithm parameters. For each one is provided: code of the Protein Data Bank and the chain used, name and number of residues for the receptor; and name, number of heavy atoms and SMILES for the ligand. The 20 structures selected for the benchmark (involving evaluation of the quality of the paths obtained) are highlighted in grey.

**PDB 1AKE (A)**	**Receptor:** Adenylate kinase (214 residues)<br>**Ligand:** Bis(adenosine)-5′-pentaphosphate (57 atoms)<br>Nc1ncnc2n(cnc12)[C@@H]1O[C@H](CO[P@@](O)(=O)O[P@@](O)(=O)OP(O)(=O)O[P@](O)(=O)O[P@](O)(=O)OC[C@H]2O[C@H]([C@H](O)[C@@H]2O)n2cnc3c(N)ncnc23)[C@@H](O)[C@H]1O
**PDB 1BBB (A)**	**Receptor:** Hemoglobin A, Alpha chain (146 residues)<br>**Ligand:** Carbon monoxide (2 atoms)<br>[C-]#[O+]
**PDB 1BBB (B)**	**Receptor:** Hemoglobin A, Beta chain (146 residues)<br>**Ligand:** Carbon monoxide (2 atoms)<br>[C-]#[O+]
**PDB 1HPU (C)**	**Receptor:** 5′-nucleotidase (525 residues)<br>**Ligand:** Phosphomethylphosphonic acid adenosyl ester (27 atoms)<br>Nc1ncnc2n(cnc12)[C@@H]1O[C@H](CO[P@](O)(=O)CP(O)(O)=O)[C@@H](O)[C@H]1O
**PDB 1MDT (A)**	**Receptor:** Diphteria toxin (535 residues)<br>**Ligand:** Adenylyl-3′-5′-phospho-uridine-3′-monophosphate (43 atoms)<br>Nc1ncnc2n(cnc12)[C@@H]1O[C@H](CO)[C@@H](O[P@@](O)(=O)OC[C@H]2O[C@H]([C@H](O)[C@@H]2OP(O)(O)=O)n2ccc(=O)[nH]c2=O)[C@H]1O
**PDB 1NCO (A)**	**Receptor:** Holo-neocarzinostatin (113 residues)<br>**Ligand:** (4R)-2-methylpentane-2,4-diol (8 atoms)<br>C[C@@H](O)CC(C)(C)O
**PDB 1NCO (B)**	**Receptor:** Holo-neocarzinostatin (113 residues)<br>**Ligand:** Neocarzinostatin-chromophore (48 atoms)<br>CN[C@H]1[C@@H](O[C@H]2[C@H](OC(=O)c3c(O)ccc4c(C)cc(OC)cc34)C=C3C#C[C@@]4(O[C@@H]4C#C\C=C2/3)[C@H]2COC(=O)O2)O[C@H](C)[C@H](O)[C@@H]1O
**PDB 1OXR (A)**	**Receptor:** Phospholipase A2 isoform 3 (119 residues)<br>**Ligand:** 2-(acetyloxy)benzoic acid (13 atoms)<br>CC(=O)Oc1ccccc1C(O)=O
**PDB 1PBO (A)**	**Receptor:** Odorant binding protein (159 residues)<br>**Ligand:** 4-butyl-5-propyl-1,3-selenazol-2-amine (13 atoms)<br>CCCCc1nc(N)[se]c1CCC
**PDB 1PV7 (A)**	**Receptor:** Lactose permease (417 residues)<br>**Ligand:** Thiodigalactoside (23 atoms)<br>OC[C@H]1O[C@@H](S[C@@H]2O[C@H](CO)[C@H](O)[C@H](O)[C@H]2O)[C@H](O)[C@@H](O)[C@H]1O
**PDB 1QFS (A)**	**Receptor:** Prolyl oligopeptidase (710 residues)<br>**Ligand:** N-benzyloxycarbonyl-L-prolyl-L-prolinal (24 atoms)<br>O=C[C@@H]1CCCN1C(=O)[C@@H]1CCCN1C(=O)OCc1ccccc1

**PDB 1T46 (A)**	**Receptor:** Homo sapiens v-kit Hardy-Zuckerman 4 feline sarcoma viral oncogene homolog (313 residues)<br>**Ligand:** 4-(4-methyl-piperazin-1-ylmethyl)-N-[4-methyl-3-(4-pyridin-3-YL-pyrimidin-2-ylamino)-phenyl]-benzamide (37 atoms)<br>CN1CCN(Cc2ccc(cc2)C(=O)Nc2ccc(C)c(Nc3nccc(n3)-c3cccnc3)c2)CC1
**PDB 1URE (A)**	**Receptor:** Intestinal fatty acid-binding protein (131 residues)<br>**Ligand:** Palmitic acid (18 atoms)<br>CCCCCCCCCCCCCCCC(O)=O
**PDB 1ZNJ**	**Receptor:** Insulin (210 residues)<br>**Ligand:** Phenol (7 atoms)<br>Oc1ccccc1
**PDB 2ACE (A)**	**Receptor:** Acetylcholinesterase (543 residues)<br>**Ligand:** Acetylcholine (10 atoms)<br>CC(=O)OCC[N+](C)(C)C
**PDB 2AMA (A)**	**Receptor:** Androgen receptor (266 residues)<br>**Ligand:** 5-alpha-dihydrotestosterone (21 atoms)<br>C[C@]12CC[C@H]3[C@@H<br>](CC[C@H]4CC(=O)CC[C@]34C)[C@@H]1CC[C@@H]2O
**PDB 2BKL (A)**	**Receptor:** Prolyl endopeptidase (695 residues)<br>**Ligand:** N-[(benzyloxy)carbonyl]-L-alanyl-L-proline (23 atoms)<br>C[C@H](NC(=O)OCc1ccccc1)C(=O)N1CCC[C@H]1C(O)=O
**PDB 2CPP (A)**	**Receptor:** Cytochrome P450-CAM (414 residues)<br>**Ligand:** Camphor (11 atoms)<br>CC1(C)[C@@H]2CC[C@@]1(C)C(=O)C2
**PDB 2DRI (A)**	**Receptor:** D-ribose-binding protein (271 residues)<br>**Ligand:** Ribose (pyranose form) (10 atoms)<br>O[C@@H]1CO[C@@H](O)[C@H](O)[C@@H]1O
**PDB 2JBV (A)**	**Receptor:** Choline oxidase (546 residues)<br>**Ligand:** [(2R,3S,4R,5R)-5-(6-amino-9H-purin-9-yl)-3,4-dihydroxytetrahydrofuran-2-yl]methyl(2R,3S,4S)-5-[(4aS,10aR)-7,8-dimethyl-2,4-dioxo-1,3,4,4a,5,10a-hexahydrobenzo[g]pteridin-10(2H)-yl]-2,3,4-trihydroxypentyl dihydrogen diphosphate (53 atoms)<br>Cc1cc2N[C@H]3C(NC(=O)NC3=O)N(C[C@H](O)[C@H](O)<br>[C@H](O)CO[P@@](O)(=O)O[P@](O)(=O)OC[C@H]3O<br>[C@H]([C@H](O)[C@@H]3O)n3cnc4c(N)ncnc34)c2cc1C
**PDB 2LBD (A)**	**Receptor:** Retinoic acid receptor gamma (267 residues)<br>**Ligand:** Retinoic acid (22 atoms)<br>C\C(\C=C\C1=C(C)CCCC1(C)C)=C/C=C/C(/C)=C/C(O)=O
**PDB 2XI4 (A)**	**Receptor:** Acetylcholinesterase (534 residues)<br>**Ligand:** Acetylcholine (10 atoms)<br>CC(=O)OCC[N+](C)(C)C
**PDB 3EBF (A)**	**Receptor:** Nitric oxide synthase (433 residues)<br>**Ligand:** (3R)-3-[(1,2,3,4-tetrahydroisoquinolin-7-yloxy)methyl]<br>-2,3-dihydrothieno[2,3-f][1,4]oxazepin-5-amine (23 atoms)<br>NC1=N[C@H](COc2ccc3CCNCc3c2)COc2ccsc12
**PDB 3FIM (A)**	**Receptor:** Aryl-alcohol oxidase (566 residues)<br>**Ligand:** Flavin-adenine dinucleotide (53 atoms)<br>Cc1cc2nc3c(nc(=O)[nH]c3=O)n(C[C@H](O)[C@H](O)[C@H](O)CO[P@](O)(=O)<br>O[P@@](O)(=O)OC[C@H]3O[C@H]([C@H](O)[C@@H]3O)n3cnc4c(N)ncnc34)c2<br>cc1C
**PDB 3GWU (A)**	**Receptor:** Leucine transporter (515 residues)<br>**Ligand:** Leucine (9 atoms)<br>CC(C)C[C@H](N)C(O)=O

**PDB 3K1O (A)**	**Receptor:** Sterol 14 alpha-demethylase (458 residues)<br>**Ligand:** 2,5-anhydro-1,3,4-trideoxy-2-(2,4-difluorophenyl)-6-O-{4-[4-(4-{1-[(1S,2S)-1-ethyl-2-hydroxypropyl]-5-oxo-1,5-dihydro-4H-1,2,4-triazol-4-yl}phenyl)piperazin-1-yl]phenyl}-1-(1H-1,2,4-triazol-1-yl)-D-erythro-hexitol (41 atoms)<br>CC[C@@H]([C@H](C)O)n1ncn(-c2ccc(cc2)N2CCN(CC2)c2ccc(OC[C@@H]3CC[C@](Cn4cncn4)(O3)c3ccc(F)cc3F)cc2)c1=O
**PDB 3NZK (A)**	**Receptor:** UDP-3-O-[3-hydroxymyristoyl] N-acetylglucosamine deacetylase (311 residues)<br>**Ligand:** N-{(1S,2R)-2-hydroxy-1-[(hydroxyamino)carbonyl]propyl}-4-{[4-(morpholin-4-ylmethyl)phenyl]ethynyl}benzamide (32 atoms)<br>C[C@@H](O)[C@H](NC(=O)c1ccc(cc1)C#Cc1ccc(CN2CCOCC2)cc1)C(=O)NO
**PDB 3O96 (A)**	**Receptor:** RAC-alpha serine/threonine-protein kinase (446 res)<br>**Ligand:** 1-(1-(4-(7-phenyl-1H-imidazo[4,5-g]quinoxalin-6-yl) benzyl)piperidin-4-yl)-1H-benzo[d]imidazol-2(3H)-one (42 atoms)<br>O=c1[nH]c2ccccc2n1C1CCN(Cc2ccc(cc2)-c2nc3cc4[nH]cnc4cc3nc2-c2ccccc2)CC1
**PDB 3PTB (A)**	**Receptor:** Beta-trypsin (223 residues)<br>**Ligand:** Benzamidine (9 atoms)<br>NC(=N)c1ccccc1
**PDB 3RUK (A)**	**Receptor:** Steroid 17-alpha-hydroxylase/17,20 lyase (494 residues)<br>**Ligand:** Abiraterone (26 atoms)<br>C[C@]12CC[C@H]3[C@@H](CC=C4C[C@@H](O)CC[C@]34C)[C@@H]1CC=C2c1cccnc1
**PDB 4GQS (A)**	**Receptor:** Cytochrome P450 2C19 (477 residues)<br>**Ligand:** (4-hydroxy-3,5-dimethylphenyl)(2-methyl-1-benzofuran-3-yl) methanone (21 atoms)<br>Cc1oc2ccccc2c1C(=O)c1cc(C)c(O)c(C)c1
**PDB 4JT6 (A)**	**Receptor:** mTOR kinase (1174 residues)<br>**Ligand:** 3-(4-morpholin-4-ylpyrido[3′,2′:4,5]furo[3,2-D]pyrimidin-2-yl) phenol (26 atoms)<br>Oc1cccc(c1)-c1nc(N2CCOCC2)c2oc3ncccc3c2n1
**PDB 4L2L (A)**	**Receptor:** Leukotriene A-4 hydrolase (611 residues)<br>**Ligand:** 4-(4-benzylphenyl)-1,3-thiazol-2-amine (19 atoms)<br>Nc1nc(cs1)-c1ccc(Cc2ccccc2)cc1
**PDB 4PYP (A)**	**Receptor:** Human glucose transporter GLUT1 (504 residues)<br>**Ligand:** B-nonylglucoside (21 atoms)<br>CCCCCCCCCO[C@@H]1O[C@H](CO)[C@@H](O)[C@H](O)[C@H]1O
**PDB 4UDC (A)**	**Receptor:** Glucocorticoid receptor (280 residues)<br>**Ligand:** Dexamethasone (28 atoms)<br>C[C@@H]1C[C@H]2[C@@H]3CCC4=CC(=O)C=C[C@]4(C)[C@@]3(F)[C@@H](O)C[C@]2(C)[C@@]1(O)C(=O)CO
**PDB 4ZW9 (A)**	**Receptor:** Human glucose transporter GLUT3 (518 residues)<br>**Ligand:** Alpha-D-glucose (12 atoms)<br>OC[C@H]1O[C@H](O)[C@H](O)[C@@H](O)[C@@H]1O
**PDB 5M0O (A)**	**Receptor:** Terminal olefin-forming fatty acid decarboxylase (422 residues)<br>**Ligand:** 5,8,11,14,17-eicosapentaenoic acid (22 atoms)<br>CC\C=C/C\C=C/C\C=C/C\C=C/C\C=C/CCCC(O)=O

**PDB 5OW9 (A)**	**Receptor:** Vitamin D3 receptor A (302 residues)<br>**Ligand:** (1~{S},3~{Z})-3-[(2~{E})-2-[(1~{S},3~{a}~{S},7~{a}~{S})-7~{a}-methyl-1-[(2~{S})-6-methyl-2-oxidanyl-heptan-2-yl]-2,3,3~{a},5,6,7-hexahydro-1~{H}-inden-4-ylidene]ethylidene]-4-methylidene-cyclohexan-1-ol (29 atoms)<br>CC(C)CCC[C@](C)(O)[C@H]1CC[C@H]2\C(CCC[C@]12C)=C\C=C1\C[C@@H](O)CCC1=C
**PDB 6ADH (A)**	**Receptor:** Holo-liver alcohol dehydrogenase (374 residues)<br>**Ligand:** Nicotinamide-adenine-dinucleotide (44 atoms)<br>NC(=O)c1ccc[n+](c1)[C@@H]1O[C@H](CO[P@]([O-])(=O)O[P@](O)(=O)OC[C@H]2O[C@H]([C@H](O)[C@@H]2O)n2cnc3c(N)ncnc23)[C@@H](O)[C@H]1O
**PDB 6MQ6 (A)**	**Receptor:** Indoleamine 2,3-dioxygenase 1 (425 residues)<br>**Ligand:** (2R)-N-(4-chlorophenyl)-2-[cis-4-(6-fluoroquinolin-4-yl)cyclohexyl]propanamide (29 atoms)<br>C[C@H]([C@H]1CC[C@H](CC1)c1ccnc2ccc(F)cc12)C(=O)Nc1ccc(Cl)cc1

Table S3. Results obtained from the GA parameters setup. Two parameters were considered: crossover/mutation proportion and "minimum increment distance from the origin". Three different values were tested for the first parameter and five values for the latter. Ten runs of calculations were carried out for each value. The average normalized clash score (being 0 the best score and 1 the worst) is reported. Best score is highlighted in grey.

PDB code	Proportion of mutation			Minimum increment distance from the origin				
	20%	50%	80%	-0.4 Å	0 Å	0.4 Å	0.6 Å	0.8 Å
1ake (a)	1,00	0,24	0,00	1,00	0,44	0,46	0,16	0,00
1bbb (a)	1,00	0,26	0,00	1,00	0,38	0,14	0,00	0,05
1bbb (b)	1,00	0,00	0,37	1,00	0,23	0,24	0,00	0,18
1hpu (c)	1,00	0,16	0,00	1,00	0,93	0,18	0,34	0,00
1mdt (a)	1,00	0,69	0,00	0,16	1,00	0,80	0,00	0,27
1nco (a)	1,00	0,00	0,29	1,00	0,45	0,04	0,20	0,00
1nco (b)	1,00	0,54	0,00	1,00	0,14	0,23	0,08	0,00
1oxr (a)	0,22	0,00	1,00	1,00	0,47	0,00	0,07	0,05
1pbo (a)	1,00	0,14	0,00	1,00	0,59	0,00	0,21	0,26
1pv7 (a)	1,00	0,00	0,13	1,00	0,18	0,00	0,04	0,40
1qfs (a)	1,00	0,52	0,00	1,00	0,85	0,26	0,02	0,00
1t46 (a)	1,00	0,01	0,00	1,00	0,88	0,23	0,00	0,49
1ure (a)	1,00	0,00	0,12	1,00	0,53	0,48	0,30	0,00
1znj	1,00	0,05	0,00	1,00	0,18	0,00	0,33	0,04
2ace (a)	1,00	0,24	0,00	1,00	0,38	0,12	0,00	0,05
2ama (a)	1,00	0,26	0,00	1,00	0,67	0,08	0,00	0,17
2bkl (a)	1,00	0,12	0,00	1,00	0,68	0,30	0,20	0,00
2cpp (a)	0,88	1,00	0,00	1,00	0,69	0,65	0,69	0,00
2dri (a)	1,00	0,00	0,07	1,00	0,51	0,21	0,23	0,00
2jbv (a)	1,00	0,59	0,00	0,77	1,00	0,48	0,17	0,00
2lbd (a)	1,00	0,40	0,00	1,00	0,71	0,49	0,01	0,00
2xi4 (a)	1,00	0,03	0,00	1,00	0,48	0,20	0,00	0,13
3ebf (a)	1,00	0,00	0,07	1,00	0,63	0,13	0,00	0,37
3fim (a)	1,00	0,00	0,10	1,00	0,36	0,49	0,24	0,00
3gwu (a)	1,00	0,00	0,41	1,00	0,00	0,13	0,87	0,18
3k1o (a)	1,00	0,00	0,06	1,00	0,86	0,80	0,00	0,45
3nzk (a)	1,00	0,24	0,00	1,00	0,10	0,38	0,00	0,04
3o96 (a)	1,00	0,00	0,21	1,00	0,98	0,12	0,20	0,00

3ptb (a)	1,00	0,00	0,04	1,00	0,31	0,06	0,03	0,00
3ruk (a)	1,00	0,54	0,00	1,00	0,43	0,10	0,21	0,00
4gqs (a)	1,00	0,34	0,00	1,00	0,74	0,13	0,00	0,59
4jt6 (a)	1,00	0,16	0,00	1,00	0,62	0,19	0,17	0,00
4l2l (a)	1,00	0,07	0,00	1,00	0,33	0,00	0,24	0,26
4pyp (a)	1,00	0,35	0,00	1,00	0,33	0,00	0,01	0,02
4udc (a)	1,00	0,00	0,24	1,00	0,95	0,85	0,89	0,00
4zw9 (a)	1,00	0,49	0,00	1,00	0,26	0,15	0,00	0,07
5m0o (a)	1,00	0,00	0,33	1,00	0,53	0,13	0,07	0,00
5ow9 (a)	1,00	0,00	0,00	1,00	0,36	0,29	0,30	0,00
6adh (a)	1,00	0,89	0,00	1,00	0,57	0,54	0,00	0,64
6mq6 (a)	1,00	0,00	0,33	0,02	1,00	0,00	0,06	0,38
**Average**	**0,96**	**0,20**	**0,12**	**0,95**	**0,54**	**0,25**	**0,16**	**0,13**

Table S4. Results obtained in the benchmark for the 20 selected systems. For each one is provided: the total number of solutions (pathways) obtained from the 20 runs of the experiment, the proportion of solutions that belong to already known pathways for that system, the proportion of solutions that belong to other pathways, a brief comment of the reference study used to obtain the well-characterized pathways and an illustrative figure.

**1BBB (A)**	**Total solutions:** 327<br>**Belonging to known pathways:** 237 (72.5%)<br>**Belonging to other pathways:** 90 (27.5%)
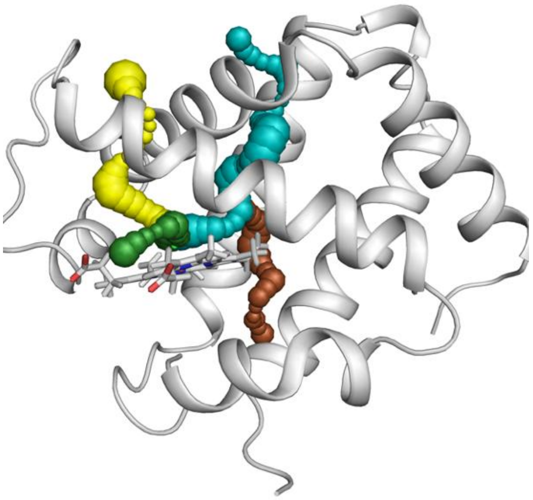	**Comments:**<br>Four main routes were identified (in green, blue, yellow and brown in the figure) for the exit of CO from human hemoglobin in a computational study [13] based on PELE [14] calculations. 72.5% of the pathways found in the benchmark correspond with one of those four.

**1OXR (A)**	**Total solutions:** 262<br>**Belonging to known pathway:** 262 (100.0%)<br>**Belonging to other pathways:** 0 (0.0%)
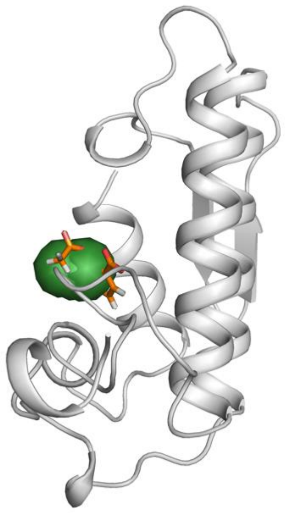	**Comments:**<br>This case is one of the examples in PELE web server [15]. Being the ligand highly solvent-exposed, it is the easiest case of the benchmark in terms of route calculation. All the solutions provided by GPathFinder are in accordance to the path proposed by PELE software.
**1QFS (A)**	**Total solutions:** 315<br>**Belonging to known pathways:** 230 (73.0%)<br>**Belonging to other pathways:** 85 (27.0%)
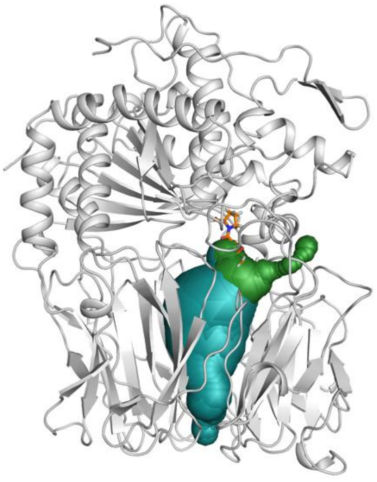	**Comments:**<br>A computational study [16] using PELE [14] identified a preference for the exit of the product using the lateral path (in green) over the pore in the beta-propeller domain (in blue). The same trend is observed in the benchmark results, with a 56.8% and 16.2% of the pathways found for these two routes, respectively.

**1T46 (A)**	**Total solutions:** 336<br>**Belonging to known pathway:** 284 (84.5%)<br>**Belonging to other pathways:** 52 (15.5%)
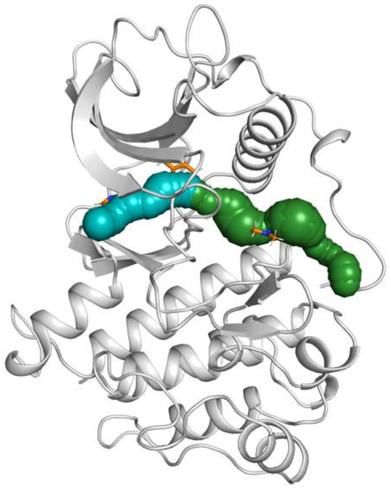	**Comments:**<br>Two channels (in green and blue) were detected to be used as binding routes in this system, which was discussed as a benchmark case in the ART-RRT article [17]. 84.5% of the solutions of GPathFinder belong to one of these two pathways.
**1URE (A)**	**Total solutions:** 267<br>**Belonging to known pathway:** 207 (77.5%)<br>**Belonging to other pathways:** 60 (22.5%)
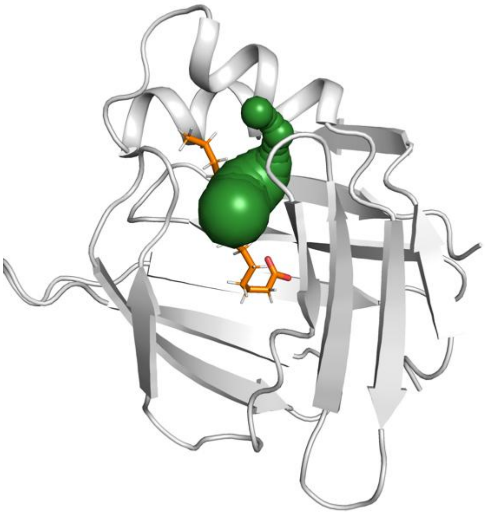	**Comments:**<br>Palmitate diffusion in the intestinal fatty-acid-binding protein was simulated as part of the representative cases in PELE article [14], identifying one exit route (in green). 77.5% of the solutions obtained in the benchmark correspond to this escape path.

**2ACE (A)**	**Total solutions:** 290<br>**Belonging to known pathways:** 225 (77.6%)<br>**Belonging to other pathways:** 65 (22.4%)
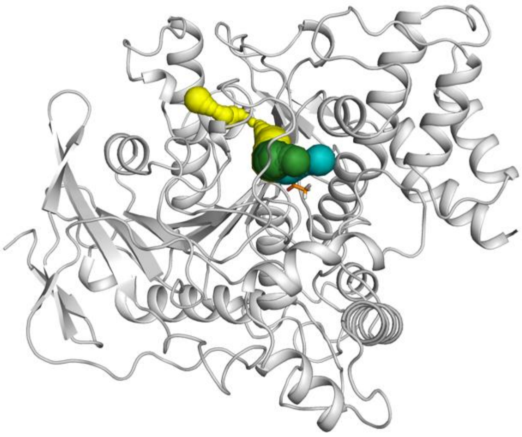	**Comments:**<br>Two main exit channels (green and blue) and a "backdoor" route (in yellow) were proposed in this illustrative case of the GRID-MD method [18]. The majority of the pathways found in GPathFinder benchmark (77.6%) correspond with one of those.
**2CPP (A)**	**Total solutions:** 348<br>**Belonging to known pathway:** 178 (51.1%)<br>**Belonging to other pathways:** 170 (48.9%)
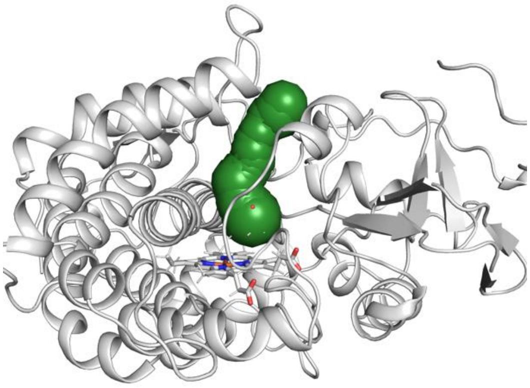	**Comments:**<br>Cytochromes P450 have a wide variety of routes for the access of ligands [19]. However, in this case study from PELE article [14], one of them (in green) was considered the one used by camphor. 51.1% of the benchmark solutions agree with this tunnel, belonging the vast majority of the rest to other known paths for P450s.

**2LBD (A)**	**Total solutions:** 301<br>**Belonging to known pathways:** 286 (95.0%)<br>**Belonging to other pathways:** 15 (5.0%)
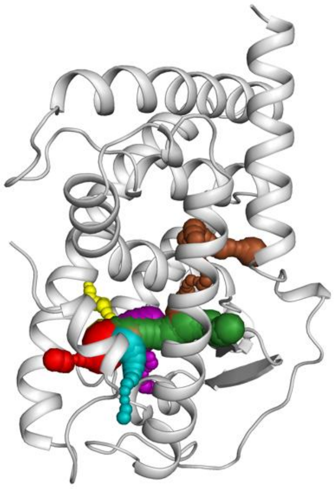	**Comments:**<br>In one of the illustrative cases of the ART-RRT paper [17], six possible routes (colored in green, blue, yellow, brown, red and purple in the figure) were proposed for this structure. 95.0% of the solutions found in the benchmark belong to one of these six.
**2XI4 (A)**	**Total solutions:** 279<br>**Belonging to "backdoor" pathway:** 122 (43.7%)<br>**Belonging to other pathways:** 157 (56.3%)
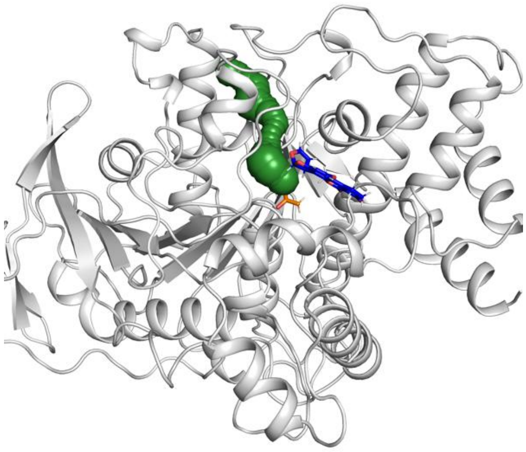	**Comments:**<br>This is the same system as seen in 2ACE, with an inhibitor (in blue sticks) blocking the main access tunnels. The ligand is expected to use the backdoor channel (in green) to access the binding site [20]. GPathFinder has been capable of identify this backdoor channel in 43.7% of the benchmark solutions.

**3EBF (A)**	**Total solutions:** 313<br>**Belonging to known pathway:** 313 (100.0%)<br>**Belonging to other pathways:** 0 (0.0%)
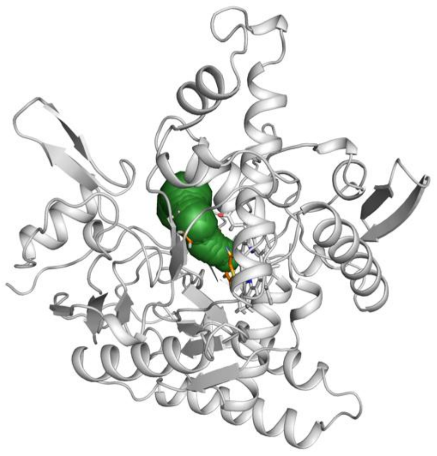	**Comments:**<br>Nitric oxide synthase has only one binding tunnel (in green) which eventually is blocked by a (selective) inhibitor [21]. In GPathFinder benchmark, the unbinding trajectory of one of such inhibitors was calculated, being the correct binding route identified in all the solutions provided by the program.
**3GWU (A)**	**Total solutions:** 313<br>**Belonging to "binding from outside" pathway:** 197 (62.9%)<br>**Belonging to other pathways:** 116 (37.1%)
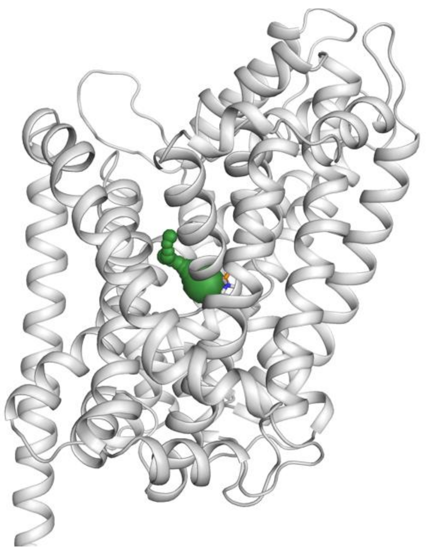	**Comments:**<br>In this case, leucine transporter is studied in complex with sertraline, an antidepressant which presumably blocks the necessary motion of the protein to release leucine inside the cell [22]. Thus, the expected routed for leucine would be the one from out the cell to the binding site (in green). 62.9% of the solutions obtained in the benchmark correspond with this path, being the rest wrong paths at which the leucine escapes through the protein laterals (space that would be occupied by other transmembrane proteins).

**3NZK (A)**	**Total solutions:** 280<br>**Belonging to known pathway:** 249 (88.9%)<br>**Belonging to other pathways:** 31 (11.1%)
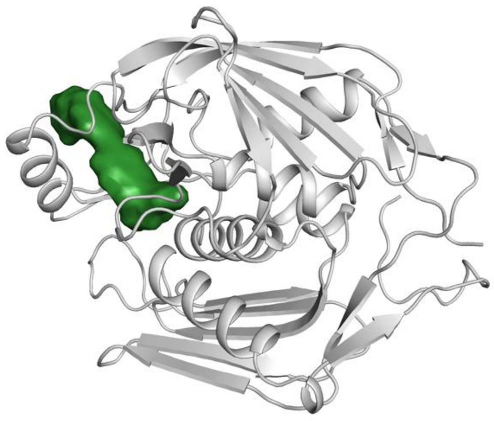	**Comments:**<br>One access tunnel (in green) is known for the binding mechanism in LpxC [23,24]. 88.9% of the solutions obtained in the benchmark correspond with this route.
**3O96 (A)**	**Total solutions:** 305<br>**Belonging to known pathway:** 167 (54.8%)<br>**Belonging to other pathways:** 138 (45.2%)
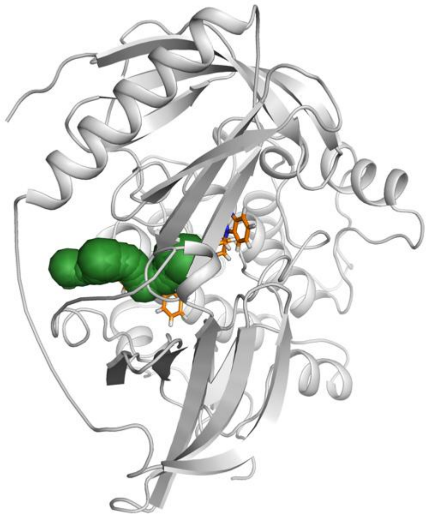	**Comments:**<br>As part of a broader study [25], the unbinding mechanism of MK-2206 was simulated with MoMA-LigPath [26], proposing the exit route highlighted in green. 54.8% of the solutions provided by GPathFinder in the benchmark agree with this path.

**3RUK (A)**	**Total solutions:** 344<br>**Belonging to known pathways:** 177 (51.5%)<br>**Belonging to other pathways:** 167 (48.5%)
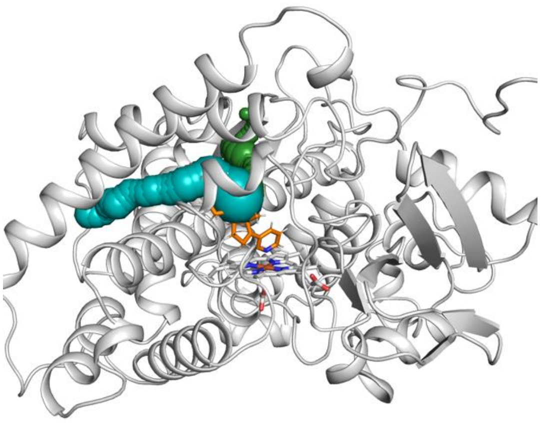	**Comments:**<br>Although cytochromes P450 have a wide variety of routes for the access of ligands [19], in this case two of them (in green and blue) were considered the dominants by a computational study [27] using MD and Caver [28]. 51.5% of the benchmark solutions belong to one of these two.
**4GQS (A)**	**Total solutions:** 247<br>**Belonging to known pathways:** 247 (100.0%)<br>**Belonging to other pathways:** 0 (0.0%)
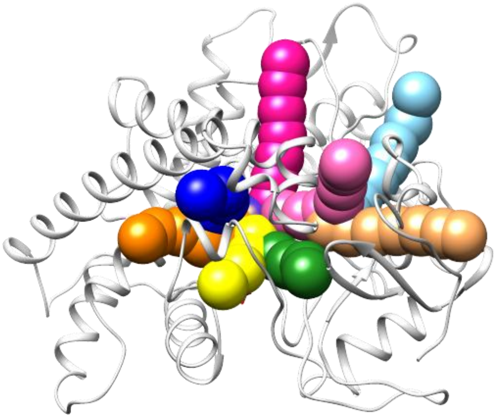	**Comments:**<br>Although the binding routes for this concrete complex have not been studied yet, a complete review of cytochromes P450 [29] provided a set of access/egress pathways, a selection of which are represented in the figure. In the benchmark results, all the solutions belong to one of these routes.

**4JT6 (A)**	**Total solutions:** 343<br>**Belonging to known pathways:** 343 (100.0%)<br>**Belonging to other pathways:** 0 (0.0%)
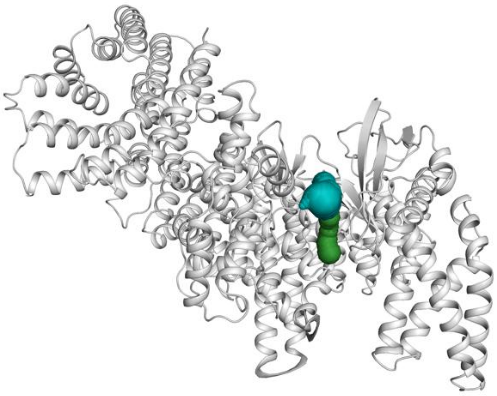	**Comments:**<br>Two binding routes (in green and blue) were proposed in a comparative study [30] for different inhibitors of the mTOR kinase, using the MoMa-LigPath software [26]. In GPathFinder benchmark, all the solutions follow one of these two paths for the native inhibitor PI-103.
**4L2L (A)**	**Total solutions:** 332<br>**Belonging to LTA_4 pathway:** 249 (75.0%)<br>**Belonging to other pathways:** 83 (25.0%)
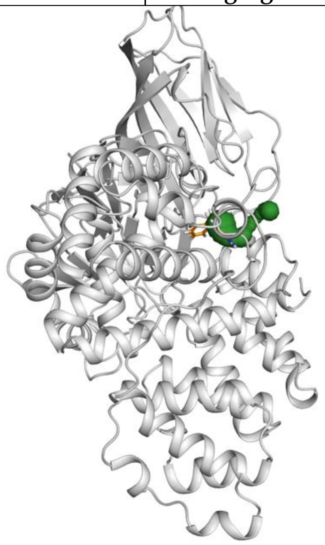	**Comments:**<br>Binding of the inhibitor ARM1 blocks the access through the tunnel used by LTA_4 to access the active site [31]. Thus, ARM1 is supposed to use this channel (in green) in its binding route. 75.0% of the solutions found in the benchmark are in agreement with this path.

**4UDC (A)**	**Total solutions:** 300<br>**Belonging to known pathway:** 257 (85.7%)<br>**Belonging to other pathways:** 43 (14.3%)
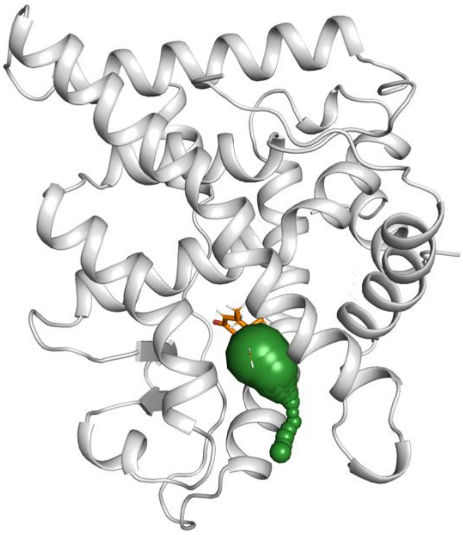	**Comments:**<br>An integral study [32] of ligand binding mechanism in steroid receptors combining experimental evidences and computational simulations with PELE [14], proposed the binding pathway shown in the figure, which correspond with 85.7% of the benchmark solutions.
**4ZW9 (A)**	**Total solutions:** 299<br>**Belonging to known pathways:** 263 (88.0%)<br>**Belonging to other pathways:** 36 (12.0%)
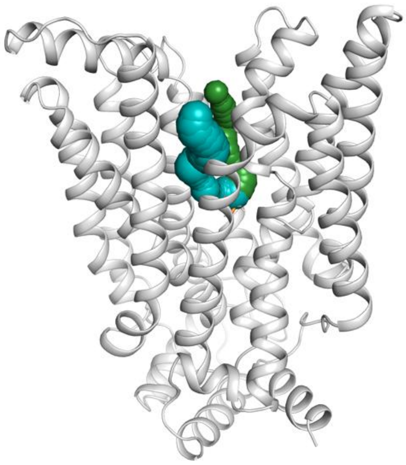	**Comments:**<br>Caver software [28] proposes two main access tunnels (in green and blue) for glucose in this glucose transporter structure. In the GPathFinder benchmark, 88.0% of the solutions found belong to one of these two routes.

**6MQ6 (A)**	**Total solutions:** 306<br>**Belonging to known pathway:** 257 (84.0%)<br>**Belonging to other pathways:** 49 (16.0%)
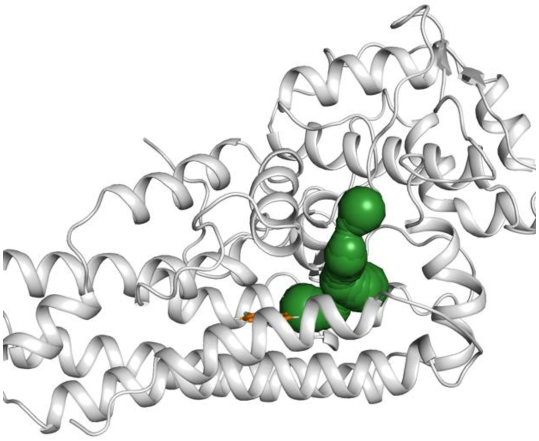	**Comments:**<br>A ligand entrance between helices K-L and N (in green) has been proposed, with some differences in the initial section of the path (see representative cases) in two experimental studies [33,34]. 84.0% of the solutions obtained in the benchmark follow this route.

3. Input *.yaml* files used in the different calculations

3.1. Input file used in the GA parameters set up calculations

```
_path: /path/to/this/input/file/param.yaml
ga:          # Section to configure the Genetic Algorithm parameters
    mut_pb: 0.8
    cx_pb: 0.2
    mut_indpb: 1
    generations: 500
    population: 12
genes:       # Section to configure the genes
-   module: gaudi.genes.molecule
    name: Ligand
    path: ./mol_files/ligand_without_H.mol2
-   module: gaudi.genes.molecule
    name: Protein
    path: ./mol_files/protein_without_H.mol2
-   module: gaudi.genes.path_torsion
    name: T
    target: Ligand
    anchor: Ligand/18 # This anchor atom is set in function of the concrete ligand
-   module: gaudi.genes.path_rotamers
    name: R
-   module: gaudi.genes.path_normalmodes
    name: NM
    target: Protein
    modes: [0,1,2,3,4,5,6,7,8,9,10,11,12,13,14,15,16,17,18,19]
    group_by: residues
    group_lambda: 15
    n_samples: 100
    write_modes: True
    write_samples: True
-   module: gaudi.genes.path
    name: Pathway
    ligand: Ligand
    protein: Protein
    torsion_gene: T
    rotamers_gene: R
    nm_gene: NM
    min_step_increment: 0.8
objectives:      # Section to configure the evaluation of the pathways
-   module: gaudi.objectives.path_scoring
    name: Clashes
    probe: Pathway
    which: clashes
    method: max
    weight: -1.0
output:
    check_every: 0
    name: param
    pareto: False
    path: /path/to/the/results/folder
similarity:
    args:
    - Pathway
    - 0.05
    kwargs: {}
```

```
    module: gaudi.path_similarity.pathways_rmsd
```

3.2. Input file used in the benchmark calculations


```
_path: /path/to/this/input/file/benchmark.yaml
ga:         # Section to configure the Genetic Algorithm parameters
    mut_pb: 0.8
    cx_pb: 0.2
    mut_indpb: 1
    generations: 750
    population: 12
genes:      # Section to configure the genes
-   module: gaudi.genes.molecule
    name: Ligand
    path: ./mol_files/ligand_with_H.mol2
-   module: gaudi.genes.molecule
    name: Protein
    path: ./mol_files/protein_with_H.mol2
-   module: gaudi.genes.path_torsion
    name: T
    target: Ligand
    anchor: Ligand/18 # This anchor atom is set in function of the concrete
ligand
-   module: gaudi.genes.path_rotamers
    name: R
-   module: gaudi.genes.path_normalmodes
    name: NM
    target: Protein
    modes: [0,1,2,3,4,5,6,7,8,9,10,11,12,13,14,15,16,17,18,19]
    group_by: residues
    group_lambda: 15
    n_samples: 100
#      forcefields:  [/path/to/protein.prmtop]  #  Used  when  non-standard
residues are present
    minimize: True
    write_modes: True
    write_samples: True
-   module: gaudi.genes.path
    name: Pathway
    ligand: Ligand
    protein: Protein
    torsion_gene: T
    rotamers_gene: R
    nm_gene: NM
    min_step_increment: 0.8
objectives:     # Section to configure the evaluation of the pathways
-   module: gaudi.objectives.path_scoring
    name: Clashes
    probe: Pathway
    which: clashes
    method: max
    weight: -1.0
-   module: gaudi.objectives.path_scoring
    name: Vina
    probe: Pathway
    which: vina
    method: max
    weight: -1.0
output:
```

```
    check_every: 0
    name: benchmark
    pareto: True
    path: /path/to/the/results/folder
similarity:
    args:
    - Pathway
    - 0.05
    kwargs: {}
    module: gaudi.path_similarity.pathways_rmsd
```


3.3. Input file used in illustrative case "aquaporins" (experiment 1)


```
_path: /path/to/this/input/file/aquaporins_clashes.yaml
ga:         # Section to configure the Genetic Algorithm parameters
    mut_pb: 0.8
    cx_pb: 0.2
    mut_indpb: 1
    generations: 500
    population: 10
genes:      # Section to configure the genes
-   module: gaudi.genes.molecule
    name: Ligand
    path: ./mol_files/ligand_without_H.mol2
-   module: gaudi.genes.molecule
    name: Protein
    path: ./mol_files/protein_without_H.mol2
-   module: gaudi.genes.path_torsion
    name: T
    target: Ligand
    anchor: Ligand/3
-   module: gaudi.genes.path_rotamers
    name: R
-   module: gaudi.genes.path_normalmodes
    name: NM
    target: Protein
    method: prody
    modes: [0,1,2,3,4,5,6,7,8,9,10,11,12,13,14,15,16,17,18,19]
    group_by: residues
    group_lambda: 15
    n_samples: 100
    rmsd: 2.0
    write_modes: True
    write_samples: True
-   module: gaudi.genes.path
    name: Pathway
    ligand: Ligand
    protein: Protein
    torsion_gene: T
    rotamers_gene: R
    nm_gene: NM
    min_step_increment: 0.8
    destination: [164.5470, 69.0624, 81.9826] # Coords of the final point
objectives:     # Section to configure the evaluation of the pathways
-   module: gaudi.objectives.path_scoring
    name: Clashes
    probe: Pathway
    which: clashes
    method: average
```

```
    weight: -1.0
output:
    check_every: 0
    name: aquaporins_clashes
    pareto: False
    path: /path/to/the/results/folder
similarity:
    args:
    - Pathway
    - 0.05
    kwargs: {}
    module: gaudi.path_similarity.pathways_rmsd
```

3.4. Input file used in illustrative case "aquaporins" (experiment 2)

```
_path: /path/to/this/input/file/aquaporins_vina.yaml
ga:         # Section to configure the Genetic Algorithm parameters
    mut_pb: 0.8
    cx_pb: 0.2
    mut_indpb: 1
    generations: 750
    population: 10
genes:      # Section to configure the genes
-   module: gaudi.genes.molecule
    name: Ligand
    path: ./mol_files/ligand_with_H.mol2
-   module: gaudi.genes.molecule
    name: Protein
    path: ./mol_files/protein_with_H.mol2
-   module: gaudi.genes.path_torsion
    name: T
    target: Ligand
    anchor: Ligand/3
-   module: gaudi.genes.path_rotamers
    name: R
-   module: gaudi.genes.path_normalmodes
    name: NM
    target: Protein
    method: prody
    modes: [0,1,2,3,4,5,6,7,8,9,10,11,12,13,14,15,16,17,18,19]
    group_by: residues
    group_lambda: 15
    n_samples: 100
    rmsd: 2.0
    minimize: True
    write_modes: True
    write_samples: True
-   module: gaudi.genes.path
    name: Pathway
    ligand: Ligand
    protein: Protein
    torsion_gene: T
    rotamers_gene: R
    nm_gene: NM
    min_step_increment: 0.8
    origin: [130.079, 67.365, 78.863]  # Coordinates of the initial point
    destination: [140.572, 68.031, 78.385]  # Coords of the final point
objectives:     # Section to configure the evaluation of the pathways
-   module: gaudi.objectives.path_scoring
```

```
    name: Clashes
    probe: Pathway
    which: clashes
    method: average
    weight: -1.0
-   module: gaudi.objectives.path_scoring
    name: Vina
    probe: Pathway
    which: vina
    method: average
    weight: -1.0
output:
    check_every: 0
    name: aquaporins_vina
    pareto: True
    path: /path/to/the/results/folder
similarity:
    args:
    - Pathway
    - 0.05
    kwargs: {}
    module: gaudi.path_similarity.pathways_rmsd
```

3.5. Input file used in illustrative case "hIDO1"

```
_path: /path/to/this/input/file/6mq6_vina.yaml
ga:         # Section to configure the Genetic Algorithm parameters
    mut_pb: 0.8
    cx_pb: 0.2
    mut_indpb: 1
    generations: 1000
    population: 32
genes:      # Section to configure the genes
-   module: gaudi.genes.molecule
    name: Ligand
    path: ./mol_files/ligand_with_H.mol2
-   module: gaudi.genes.molecule
    name: Protein
    path: ./mol_files/protein_with_H.mol2
-   module: gaudi.genes.path_torsion
    name: T
    target: Ligand
    anchor: Ligand/4
    rotatable_atom_types: []
    rotatable_atom_names: [C5,C1,N1]
-   module: gaudi.genes.path_rotamers
    name: R
-   module: gaudi.genes.path_normalmodes
    name: NM
    target: Protein
    method: prody
    modes: [0,1,2,3,4,5,6,7,8,9,10,11,12,13,14,15,16,17,18,19]
    group_by: residues
    group_lambda: 15
    n_samples: 100
    rmsd: 2.0
    minimize: True
    write_modes: True
    write_samples: True
```

```
-   module: gaudi.genes.path
    name: Pathway
    ligand: Ligand
    protein: Protein
    torsion_gene: T
    rotamers_gene: R
    nm_gene: NM
    min_step_increment: 0.8
objectives:     # Section to configure the evaluation of the pathways
-   module: gaudi.objectives.path_scoring
    name: Clashes
    probe: Pathway
    which: clashes
    method: average
    weight: -1.0
-   module: gaudi.objectives.path_scoring
    name: Vina
    probe: Pathway
    which: vina
    method: average
    weight: -1.0
output:
    check_every: 0
    name: 6mq6_vina
    pareto: True
    path: /path/to/the/results/folder
similarity:
    args:
    - Pathway
    - 0.05
    kwargs: {}
    module: gaudi.path_similarity.pathways_rmsd
```


3.6. Input file used in illustrative case "P450" (experiment 1)


```
_path: /path/to/this/input/file/4gqs_clashes.yaml
ga:         # Section to configure the Genetic Algorithm parameters
    mut_pb: 0.8
    cx_pb: 0.2
    mut_indpb: 1
    generations: 500
    population: 12
genes:      # Section to configure the genes
-   module: gaudi.genes.molecule
    name: Ligand
    path: ./mol_files/ligand_with_H.mol2
-   module: gaudi.genes.molecule
    name: Protein
    path: ./mol_files/protein_with_H.mol2
-   module: gaudi.genes.path_torsion
    name: T
    target: Ligand
    anchor: Ligand/11
-   module: gaudi.genes.path_rotamers
    name: R
-   module: gaudi.genes.path_normalmodes
    name: NM
    target: Protein
    method: prody
```

```
    modes: [0,1,2,3,4,5,6,7,8,9,10,11,12,13,14,15,16,17,18,19]
    group_by: residues
    group_lambda: 15
    n_samples: 100
    rmsd: 2.0
    minimize: True
    forcefields: [/path/to/protein.prmtop]
    write_modes: True
    write_samples: True
-   module: gaudi.genes.path
    name: Pathway
    ligand: Ligand
    protein: Protein
    torsion_gene: T
    rotamers_gene: R
    nm_gene: NM
    min_step_increment: 0.8
objectives:     # Section to configure the evaluation of the pathways
-   module: gaudi.objectives.path_scoring
    name: Clashes
    probe: Pathway
    which: clashes
    method: max
    weight: -1.0
output:
    check_every: 0
    name: 4gqs_clashes
    pareto: False
    path: /path/to/the/results/folder
similarity:
    args:
    - Pathway
    - 0.05
    kwargs: {}
    module: gaudi.path_similarity.pathways_rmsd
```

3.7. Input file used in illustrative case "P450" (experiment 2)

```
_path: /path/to/this/input/file/4gqs_vina.yaml
ga:         # Section to configure the Genetic Algorithm parameters
    mut_pb: 0.8
    cx_pb: 0.2
    mut_indpb: 1
    generations: 750
    population: 12
genes:      # Section to configure the genes
-   module: gaudi.genes.molecule
    name: Ligand
    path: ./mol_files/ligand_with_H.mol2
-   module: gaudi.genes.molecule
    name: Protein
    path: ./mol_files/protein_with_H.mol2
-   module: gaudi.genes.path_torsion
    name: T
    target: Ligand
    anchor: Ligand/11
-   module: gaudi.genes.path_rotamers
    name: R
-   module: gaudi.genes.path_normalmodes
```

```
    name: NM
    target: Protein
    method: prody
    modes: [0,1,2,3,4,5,6,7,8,9,10,11,12,13,14,15,16,17,18,19]
    group_by: residues
    group_lambda: 15
    n_samples: 100
    rmsd: 2.0
    minimize: True
    forcefields: [/path/to/protein.prmtop]
    write_modes: True
    write_samples: True
-   module: gaudi.genes.path
    name: Pathway
    ligand: Ligand
    protein: Protein
    torsion_gene: T
    rotamers_gene: R
    nm_gene: NM
    min_step_increment: 0.8
objectives:     # Section to configure the evaluation of the pathways
-   module: gaudi.objectives.path_scoring
    name: Clashes
    probe: Pathway
    which: clashes
    method: max
    weight: -1.0
-   module: gaudi.objectives.path_scoring
    name: Vina
    probe: Pathway
    which: vina
    method: max
    weight: -1.0
output:
    check_every: 0
    name: 4gqs_vina
    pareto: True
    path: /path/to/the/results/folder
similarity:
    args:
    - Pathway
    - 0.05
    kwargs: {}
    module: gaudi.path_similarity.pathways_rmsd
```

References

1. Bakan, A.; Meireles, L.M.; Bahar, I. ProDy: Protein dynamics inferred from theory and experiments. *Bioinformatics* **2011**, *27*, 1575–1577.
2. Eastman, P.; Swails, J.; Chodera, J.D.; McGibbon, R.T.; Zhao, Y.; Beauchamp, K.A.; Wang, L.P.; Simmonett, A.C.; Harrigan, M.P.; Stern, C.D.; et al. OpenMM 7: Rapid development of high performance algorithms for molecular dynamics. *PLoS Comput. Biol.* **2017**, *13*, 1–17.
3. Hornak, V.; Abel, R.; Okur, A.; Strockbine, B.; Roitberg, A.; Simmerling, C. Comparison of multiple amber force fields and development of improved protein backbone parameters. *Proteins* **2006**, *65*, 712–725.
4. Pettersen, E.F.; Goddard, T.D.; Huang, C.C.; Couch, G.S.; Greenblatt, D.M.; Meng, E.C.; Ferrin, T.E. UCSF Chimera - A visualization system for exploratory research and analysis. *J. Comput. Chem.* **2004**, *25*, 1605–1612.
5. Eyal, E.; Najmanovich, R.; Mcconkey, B.J.; Edelman, M.; Sobolev, V. Importance of Solvent Accessibility

and Contact Surfaces in Modeling Side-Chain Conformations in Proteins. *J. Comput. Chem.* **2004**, *25*, 712–724.

6. Frisch, M. J.; Trucks, G. W.; Schlegel, H. B.; Scuseria, G.E..; Robb, G. E.; Cheeseman, J. R.; Scalmani, G.; Barone, V.; Mennucci, B..; Petersson, G. A.; Nakatsuji, H.; Caricato, M.; Li, X.; Hratchian, H.P..; Izmaylov, A. F.; Bloino, J.; Zheng, G.; Sonnenberg, J. L.; Hada, M..; Ehara, M.; Toyota, K.; Fukuda, R.; Hasegawa, J.; Ishida, M.; Nakajima, T..; Honda, Y.; Kitao, O.; Nakai, H.; Vreven, T.; Montgomery, Jr., J.A.. P.; J. E.; Ogliaro, F.; Bearpark, M.; Heyd, J. J.; Brothers, E.. K.; K. N.; Staroverov, V. N.; Keith, T.; Kobayashi, R.; Normand, J.. R.; K.; Rendell, A.; Burant, J. C.; Iyengar, S. S.; Tomasi, J.. C.; M.; Rega, N.; Millam, J. M.; Klene, M.; Knox, J. E.; Cross, J.B..; et al. Gaussian 09, Revision D.01. *Gaussian Inc.* 2009.
7. Ehlers, A.W.; Böhme, M.; Dapprich, S.; Gobbi, A.; Höllwarth, A.; Jonas, V.; Köhler, K.F.; Stegmann, R.; Veldkamp, A.; Frenking, G. A set of f-polarization functions for pseudo-potential basis sets of the transition metals ScCu, YAg and LaAu. *Chem. Phys. Lett.* **1993**, *208*, 111–114.
8. Bayly, C.I.; Cieplak, P.; Cornell, W.D.; Kollman, P.A. A well-behaved electrostatic potential based method using charge restraints for deriving atomic charges: The RESP model. *J. Phys. Chem.* **1993**, *97*, 10269–10280.
9. Li, P.; Merz, K.M. MCPB.py: A Python Based Metal Center Parameter Builder. *J. Chem. Inf. Model.* **2016**.
10. Lavalle, S.M. *Rapidly-Exploring Random Trees: A New Tool for Path Planning*; Iowa, 1998;
11. Cortés, J.; Jaulet, L.; Siméon, T. Molecular disassembly with rrt-like algorithms. In Proceedings of the IEEE International Conference on Robotics and Automation; Roma, 2007; pp. 3301–3306.
12. Kuffner, J.J.; LaValle, S.M. RRT-connect: An efficient approach to single-query path planning. In Proceedings of the 2000 IEEE International Conference on Robotics & Automation; Institute of Electrical and Electronics Engineers (IEEE): San Francisco, 2000; pp. 995–1001.
13. Lucas, M.F.; Guallar, V. An atomistic view on human hemoglobin carbon monoxide migration processes. *Biophys. J.* **2012**, *102*, 887–896.
14. Borrelli, K.W.; Vitalis, A.; Alcantara, R.; Guallar, V. PELE: Protein energy landscape exploration. A novel Monte Carlo based technique. *J. Chem. Theory Comput.* **2005**, *1*, 1304–1311.
15. Madadkar-Sobhani, A.; Guallar, V. PELE web server: atomistic study of biomolecular systems at your fingertips. *Nucleic Acids Res.* **2013**, *41*, W332–W328.
16. Kotev, M.; Lecina, D.; Tarragó, T.; Giralt, E.; Guallar, V. Unveiling prolyl oligopeptidase ligand migration by comprehensive computational techniques. *Biophys. J.* **2015**, *108*, 116–125.
17. Nguyen, M.K.; Jaillet, L.; Redon, S. ART-RRT: As-Rigid-As-Possible exploration of ligand unbinding pathways. *J. Comput. Chem.* **2018**, *39*, 665–678.
18. Carrillo, O.; Orozco, M. GRID-MD-A tool for massive simulation of protein channels. *Proteins Struct. Funct. Genet.* **2008**, *70*, 892–899.
19. Cojocaru, V.; Winn, P.J.; Wade, R.C. The ins and outs of cytochrome P450s. *Biochim. Biophys. Acta - Gen. Subj.* **2007**, *1170*, 390–401.
20. Sanson, B.; Colletier, J.P.; Xu, Y.; Lang, P.T.; Jiang, H.; Silman, I.; Sussman, J.L.; Weik, M. Backdoor Opening Mechanism in Acetylcholinesterase Based on X-Ray Crystallography and Molecular Dynamics Simulations. *Protein Sci.* **2011**, *20*, 1114–1118.
21. Garcin, E.D.; Arvai, A.S.; Rosenfeld, R.J.; Kroeger, M.D.; Crane, B.R.; Andersson, G.; Andrews, G.; Hamley, P.J.; Mallinder, P.R.; Nicholls, D.J.; et al. Anchored plasticity opens doors for selective inhibitor design in nitric oxide synthase. *Nat. Chem. Biol.* **2008**, *4*, 700–707.
22. Zhou, Z.; Zhen, J.; Karpowich, N.K.; Law, C.J.; Reith, M.E.; Wang, D.N. Antidepressant specificity of serotonin transporter suggested by three LeuT-SSRI structures. *Nat.Struct.Mol.Biol.* **2009**, *16*, 652–657.
23. Cole, K.E.; Gattis, S.G.; Angell, H.D.; Fierke, C.A.; Christianson, D.W. Structure of the Metal-Dependent Deacetylase LpxC from Yersinia enterocolitica Complexed with the Potent Inhibitor CHIR-090 . *Biochemistry* **2010**, *50*, 258–262.
24. Barb, A.W.; Zhou, P. Mechanism and inhibition of LpxC: an essential zinc-dependent deacetylase of bacterial lipid A synthesis. *Curr. Pharm. Biotechnol.* **2008**, *9*, 9–15.
25. Rehan, M.; Beg, M.A.; Parveen, S.; Damanhouri, G.A.; Zaher, G.F. Computational insights into the inhibitory mechanism of human AKT1 by an orally active inhibitor, MK-2206. *PLoS One* **2014**, *9*, 1–12.
26. Devaurs, D.; Bouard, L.; Vaisset, M.; Zanon, C.; Al-Bluwi, I.; Iehl, R.; Siméon, T.; Cortés, J. MoMA-LigPath: a web server to simulate protein-ligand unbinding. *Nucleic Acids Res.* **2013**, *41*, W297–W302.
27. Cui, Y.L.; Zheng, Q.C.; Zhang, J.L.; Xue, Q.; Wang, Y.; Zhang, H.X. Molecular dynamic investigations of the mutational effects on structural characteristics and tunnel geometry in CYP17A1. *J. Chem. Inf. Model.*

2013, *53*, 3308–3317.

28. Chovancova, E.; Pavelka, A.; Benes, P.; Strnad, O.; Brezovsky, J.; Kozlikova, B.; Gora, A.; Sustr, V.; Klvana, M.; Medek, P.; et al. CAVER 3.0: A Tool for the Analysis of Transport Pathways in Dynamic Protein Structures. *PLoS Comput. Biol.* **2012**, *8*, 1–12.
29. Mustafa, Ghulam; Yu, Xiaofeng; Wade, R.C. Structure and dynamics of human drug-metabolizing cytochrome P450 enzymes. In *Drug metabolism prediction*; Kirchmair, J., Ed.; Wiley-VCH: Weinheim, 2014; pp. 77–101.
30. Rehan, M. An Anti-Cancer Drug Candidate OSI-027 and its Analog as Inhibitors of mTOR: Computational Insights Into the Inhibitory Mechanisms. *J. Cell. Biochem.* **2017**, *118*, 4558–4567.
31. Stsiapanava, A.; Olsson, U.; Wan, M.; Kleinschmidt, T.; Rutishauser, D.; Zubarev, R.A.; Samuelsson, B.; Rinaldo-Matthis, A.; Haeggstrom, J.Z. Binding of Pro-Gly-Pro at the active site of leukotriene A4 hydrolase/aminopeptidase and development of an epoxide hydrolase selective inhibitor. *Proc. Natl. Acad. Sci.* **2014**, *111*, 4227–4232.
32. Edman, K.; Hosseini, A.; Bjursell, M.K.; Aagaard, A.; Wissler, L.; Gunnarsson, A.; Kaminski, T.; Köhler, C.; Bäckström, S.; Jensen, T.J.; et al. Ligand Binding Mechanism in Steroid Receptors: From Conserved Plasticity to Differential Evolutionary Constraints. *Structure* **2015**, *23*, 2280–2290.
33. Pham, K.N.; Yeh, S.-R. Mapping the Binding Trajectory of a Suicide Inhibitor in Human Indoleamine 2,3-Dioxygenase 1. *J. Am. Chem. Soc.* **2018**, *140*, 14538–14541.
34. Sugimoto, H.; Oda, S. -i.; Otsuki, T.; Hino, T.; Yoshida, T.; Shiro, Y. Crystal structure of human indoleamine 2,3-dioxygenase: Catalytic mechanism of O2 incorporation by a heme-containing dioxygenase. *Proc. Natl. Acad. Sci.* **2006**, *103*, 2611–2616.

© 2019 by the authors. Submitted for possible open access publication under the terms and conditions of the Creative Commons Attribution (CC BY) license (http://creativecommons.org/licenses/by/4.0/).
